# Micro-scale opto-thermo-mechanical actuation in the dry adhesive regime

**DOI:** 10.1038/s41377-021-00622-6

**Published:** 2021-09-22

**Authors:** Weiwei Tang, Wei Lyu, Jinsheng Lu, Fengjiang Liu, Jiyong Wang, Wei Yan, Min Qiu

**Affiliations:** 1 Key Laboratory of 3D Micro/Nano Fabrication and Characterization of Zhejiang Province, School of Engineering, Westlake University, 18 Shilongshan Road, Hangzhou, 310024 Zhejiang Province China; 2 Institute of Advanced Technology, Westlake Institute for Advanced Study, 18 Shilongshan Road, Hangzhou, 310024 Zhejiang Province China; 3 State Key Laboratory of Modern Optical Instrumentation, College of Optical Science and Engineering, Zhejiang University, Hangzhou, 310027 China

**Keywords:** Optical manipulation and tweezers, Nanophotonics and plasmonics

## Abstract

Realizing optical manipulation of microscopic objects is crucial in the research fields of life science, condensed matter physics, and physical chemistry. In non-liquid environments, this task is commonly regarded as difficult due to strong adhesive surface force (~µN) attached to solid interfaces that makes tiny optical driven force (~pN) insignificant. Here, by recognizing the microscopic interaction mechanism between friction force—the parallel component of surface force on a contact surface—and thermoelastic waves induced by pulsed optical absorption, we establish a general principle enabling the actuation of micro-objects on dry frictional surfaces based on the opto-thermo-mechanical effects. Theoretically, we predict that nanosecond pulsed optical absorption with mW-scale peak power is sufficient to tame µN-scale friction force. Experimentally, we demonstrate the two-dimensional spiral motion of gold plates on micro-fibers driven by nanosecond laser pulses, and reveal the rules of motion control. Our results pave the way for the future development of micro-scale actuators in non-liquid environments.

## Abstract (web summary)

A general principle enabling the actuation of micro-objects on dry frictional surfaces based on opto-thermo-mechanical effects is established and two-dimensional spiral motions of gold plates on micro-fibers are experimentally demonstrated.

## Graphical abstract

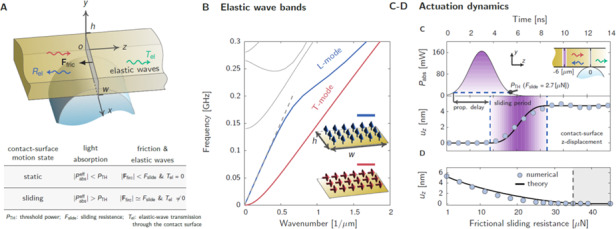

## Introduction

In his landmark lecture “There is plenty of room at the bottom” in 1959, Richard Feynman envisioned a fundamental scaling challenge in the coming era of nanotechnology: macroscopic designing rules for mechanical devices shall become invalid at micro-scales where adhesive surface force plays a dominant role in the mechanical response of devices as a result of increased surface-to-volume ratio^1^. To bypass this challenge, the micro-scale mechanical devices, especially actuators—indispensable in various applications, such as optical communications^2–5^, optical displays^6^ and molecular cargo^7,8^—, nowadays generally exploit membrane architecture to mitigate undesirable surface effects and employ scale-invariant electrostatic force^9,10^. Alternatively, they operate in low adhesive environments (e.g., immersed in liquids) using tiny optical force (~pN)^11–17^, photoelectric force (~ pN)^17–19^ or taking advantage of bio-inspired actuation strategy^20^. Realizing micro-scale actuators that can freely walk on two-dimensional dry (non-liquid) surfaces directly against strong resistance from the parallel component of surface force, i.e., friction force (~µN), is challenging^21^.

In this regard, a few recent studies have pointed out a new direction: the excitations of elastic waves in micro-objects could facilitate their locomotion on dry surfaces^22–24^, as an extension of macroscopic surface elastic-wave motors^25,26^. In these pioneering studies, elastic waves are excited by temperature changes in micro-objects through optical absorption based on fiber-based systems. Essentially, this is different from another scheme widely adopted in micro-fluidics, wherein elastic waves are generated by the electronic excitations with piezoelectric substrates and are used to drive the locomotion of cells, droplets, particles, etc. for lab-on-a-chip technologies^27^. Besides, the findings in these studies have demonstrated motions of specific-shaped gold plates either along the azimuthal^23^ or axial directions^22,24^ of the micro-fibers, but not both.

However, these existing results severely lack a convincing theory—that takes into account of the fundamental role of friction force at micro-scales—to inspire new designs, and experimentally only access to the one-dimensional motion. Here we resolve all these limitations both theoretically and experimentally. A theory that takes microscopic interactions between friction force and thermally excited elastic waves into account—supplemented with an illustrative case study—is established. It features a predictive equation for the threshold optical power needed to overcome friction resistance. The double role of friction force in both hindering and promoting actuation is explicitly revealed. Experimentally, we observe two-dimensional spiral motions of gold plates with various shapes (including triangle, rectangle, square, hexagon, and circle) on micro-fibers driven by a nanosecond pulsed laser, and demonstrate, validate the general rules of motion control. Further, we provide the direct observation of the threshold optical power of motion, thereby validating the proposed theory. These results advantageously provide a solid framework for understanding, interpreting, modeling the actuation dynamics involving friction force at micro-scales and for further inspiring new designs of miniature actuators based on similar principles.

## Results

### Principle: interplay between friction force and elastic waves

**Fig. 1:**
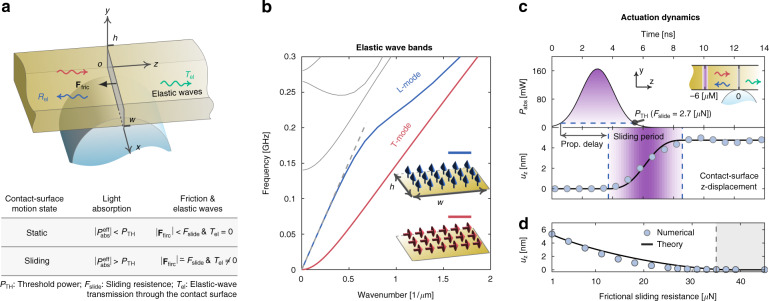
Principle of opto-thermo-mechanical actuation overcoming µN-scale friction force at micro-scales. **a** Driving locomotion of a rectangular plate on a frictional surface through excitations of elastic waves by pulsed optical absorption. The contact surface is a line-shaped region rendered by the surface curvature of the substrate. Table: relationships between motion states of the plate, (instantaneous) effective absorbed light power, friction force and elastic waves. **b** Band structure of elastic waveguide modes in a gold plate (width, *w* = 4 µm; height, *h* = 60 nm). Insets: modal profiles of fundamental longitudinal (*L*)- and transverse (*T*)-like modes at static frequency (arrows specify directions of elastic oscillations). **c** Sliding displacement of the contact surface of the gold plate (same as in **b**; lower panel) in the *z*-direction driven by a nanosecond optical pulse (upper panel) with frictional sliding resistance F_slide _= 2.7 µN. The absorbed optical power distributes uniformly in the *x-y* plane, has a Gaussian width of 1 µm in the *z*-direction and localizes on the left side of the contact surface (sketched in the inset of the upper panel). **d** Stabilized sliding displacement as a function of sliding resistance, F_slide_. In (**c**, **d**), the numerical results are obtained with the COMSOL Multiphysics (circles) and agree with the theoretical predictions [solid lines; computed with Eq. (2.18) in the Supplementary Material]. The elastic attenuation is ignored

We start with a case study to pedagogically interpret the principle of the proposed micro-scale opto-thermo-mechanical actuation scheme and to clarify the key role of elastic waves—excited by mild (mW-scale) optical absorption—in overcoming friction force. As shown in Fig. 1a, a gold rectangular plate is placed on a curved substrate. The contact surface of the two structures is a line-shaped region, resembling the later demonstrated experiment. Illuminated by laser pulses, the plate absorbs light and then converts it to heat that induces lattice oscillations, i.e., elastic waves^28^. The excited elastic waves intend to drive the plate to deform its original shape, which, however, is resisted by the friction force on the contact surface^29^. Thereupon, the elementary physical process here lies in the interaction between incident elastic waves—that propagate toward the contact surface from one side—and the friction force (sketched in Fig. 1a), which we shall discuss below.

We first examine the excited elastic waves in the plate by representing them with waveguide modes. Fig. 1b shows the band structure of these modes (propagating in the *z*-direction) supported by a rectangular plate with cross-sectional width *w* = 4 µm and thickness *h* = 60 nm. Among a variety of modes, we highlight the fundamental longitudinal (*L*)- and transverse (*T*)-like modes (see Sec. 1 in the Supplementary Material for the derivations of their dispersion relations at low frequencies), which show no cutoff, and oscillate parallel and perpendicular to their propagation directions, respectively (see the modal profiles in the insets of Fig. 1b). These modes are important since they are dominantly excited, e.g., by nanosecond laser pulses (as in our experiment), and moreover their static-frequency contributions determine the stabilized locomotion state of the plate along the longitudinal (*z*-) and transverse (*x*-) directions, respectively.

From a microscopic wave picture, the friction force behaves as a fence resisting the elastic waves from passing through the contact surface (see the table in Fig. 1a, and Fig. S5 in the Supplementary Material for numerical illustrations). In the static regime, the static friction force is large enough to totally reflect back the incident elastic waves and thus nullify mechanical oscillations on the contact surface (that is, the incident and reflected elastic waves cancel each other out perfectly), thereby preserving the contact surface in the still state. On the contrary, in the dynamic regime, the elastic waves are so strong that even the maximum allowable static friction force—the so-called sliding resistance force denoted by ${\rm{F}}_{{{{\mathrm{slide}}}}}$—cannot nullify them on the contact surface. The plate thus slides.

Apparently, the static-to-sliding transition occurs at a critical point when the induced elastic waves due to the sliding resistance ${{{\mathrm{F}}}}_{{{{\mathrm{slide}}}}}$ perfectly cancel with the incident elastic waves due to optical absorption. In view of this, we introduce the sliding threshold power ${{{\mathrm{P}}}}_{{{{\mathrm{TH}}}}}$—the minimum of the magnitude of the instantaneous effective absorbed optical power, ${{{\mathrm{P}}}}_{{{{\mathrm{abs}}}}}^{{{{\mathrm{eff}}}}}$, required to overcome the sliding resistance—to quantify the power cost of this transition. Here, the effective absorbed optical power is defined by ${{{\mathrm{P}}}}_{{{{\mathrm{abs}}}}}^{{{{\mathrm{eff}}}}} = {{{\mathrm{P}}}}_{{{{\mathrm{abs}}}}} - {{{\mathrm{P}}}}_{{{{\mathrm{leak}}}}}$, i.e., the absorbed optical power ${{{\mathrm{P}}}}_{{{{\mathrm{abs}}}}}$ minus the leaked power ${{{\mathrm{P}}}}_{{{{\mathrm{leak}}}}}$. For simplicity of analysis, we assume that the optical absorption—that induces the incident elastic waves—uniformly distributes along the oscillation direction of the *T*-modes (*x*-direction), so that only the sliding along the longitudinal direction (*z*-direction) relating to the *L*-modes is activated. The sliding threshold power is then interpreted as the magnitude of the effective absorbed optical power that together with the sliding resistance results in zero amplitude of the *L*-modes on the contact surface. It is derived as (see Sec. 2 in the Supplementary Material)

1 $${{{\mathrm{P}}}}_{{{{\mathrm{TH}}}}} \cong \frac{{c_p{{{\mathrm{F}}}}_{{{{\mathrm{slide}}}}}}}{{\alpha _{{{{\mathrm{th}}}}}\upsilon _{3{{{\mathrm{D}}}}}^{{{\mathrm{L}}}}}}e^{t_0/\tau _{{{{\mathrm{ac}}}}}}$$

Here, $c_p$ and $\alpha _{{{{\mathrm{th}}}}}$ denote the specific heat capacity and the coefficient of linear thermal expansion of gold, respectively. $\upsilon _{3{{{\mathrm{D}}}}}^{{{\mathrm{L}}}} = \sqrt {E/\rho } \cong 2000\,m/s$ ($E$ and $\rho$ denoting Young’s modulus and density of gold, respectively) is the velocity of the *L*-modes at low frequencies, wherein a linear dispersion relation emerges [see the dashed line in Fig. 1b and Eq. (1.6a) in the Supplementary Material]. $t_0$ is the time for elastic waves traveling from the center of the absorbed optical power to the contact surface and $\tau _{{{{\mathrm{ac}}}}}$ is the life time of the elastic waves ^30,31^.

### Implications of threshold power

We now discuss the dependence of the threshold power ${{{\mathrm{P}}}}_{{{{\mathrm{TH}}}}}$ on the dimension of the plate. First, the term of the sliding resistance in Eq. (1), ${{{\mathrm{F}}}}_{{{{\mathrm{slide}}}}}$, renders ${{{\mathrm{P}}}}_{{{{\mathrm{TH}}}}}$ a linear dependence on the width of the plate along the *x*-axis, i.e., *w*. Specially, ${{{\mathrm{F}}}}_{{{{\mathrm{slide}}}}}$ linearly scales with the area of the contact surface^29^, which—in our case study—is proportional to *w*. Second, we observe that the threshold power increases with the propagation time of the elastic waves $t_0$, which indirectly associates with the width of the plate along the *z*-axis (that is, the larger of this width, potentially the longer $t_0$). Finally, we point out that the thickness dependence is absent in our theory, which holds as long as the thickness of the plate is much smaller than the elastic wavelength of interest (see Sec. 2 in the Supplementary Material).

Equation (1) signifies the feasibility of the actuation of the plate by using practical optical power. Specifically, P_TH_ ≅ F_slide_ × 2mW × μN for gold (neglecting elastic attenuation; see Table I in the Supplementary Material for thermal and mechanical parameters of gold), implying that mW-scale effective absorbed optical power is sufficient to overcome µN-scale friction force. Such mild instantaneous power can be readily realized with micro-/nano- structures made of gold—and also a large variety of other lossy materials—with broadband losses, and by additionally exploiting resonance effects (e.g., plasmonic excitations and Mie’s resonances)^32,33^. More importantly, regarding laser sources, Eq. (1) guides that *pulsed lasers are the ideal choice for the proposed actuation scheme*, featuring two unique advantages: (i) high peak power facilitating the effective absorbed instantaneous power to surpass ${{{\mathrm{P}}}}_{{{{\mathrm{TH}}}}}$ and (ii) low single-pulse energy and relatively long repetition period benefiting thermal cooling.

Equation (1) also implies that continuous wave (CW) lasers are deficient for the proposed actuation scheme. This is because that, with the CW lasers, the plate will rise to a constant temperature having ${{{\mathrm{P}}}}_{{{{\mathrm{abs}}}}} = {{{\mathrm{P}}}}_{{{{\mathrm{leak}}}}}$, which leads to ${{{\mathrm{P}}}}_{{{{\mathrm{abs}}}}}^{{{{\mathrm{eff}}}}} = 0$. Accordingly, the threshold power ${{{\mathrm{P}}}}_{{{{\mathrm{TH}}}}}$ is failed to be overcome.

Employing pulsed lasers, the plate slides within the period when the magnitude of the effective absorbed optical power is above ${{{\mathrm{P}}}}_{{{{\mathrm{TH}}}}}$. To straightforwardly reveal these actuation dynamics, we perform numerical simulations by considering that a gold rectangular plate—with width *w* = 4 µm and height *h* = 60 nm (same as in Fig. 1b)—is driven by nanosecond pulsed absorbed optical power. The plate is placed on a curved cylindrical surface, and the friction resistance between them is ${{{\mathrm{F}}}}_{{{{\mathrm{slide}}}}}$ = 2.7 µN. Consequently, the threshold power predicted with Eq. (1) is about ${{{\mathrm{P}}}}_{{{{\mathrm{TH}}}}} \cong 5.4\,{{{\mathrm{mW}}}}$. We set the peak power of the optical absorption to be 160 mW (see the upper panel of Fig. 1c for its temporal profile), which exceeds ${{{\mathrm{P}}}}_{{{{\mathrm{TH}}}}}$, and additionally ensures that the maximum rising temperature of the plate (about $600^\circ {{{\mathrm{C}}}}$) is well below the melting temperature of gold. Besides, the absorbed optical power, which localizes in a line-shaped region (see the caption of Fig. 1 for more details), is set to be 6 µm away from the contact surface in the negative the *z* direction, from where the thermally excited elastic waves impinge on the contact surface.

As is shown in the lower panel of Fig. 1c, the sliding takes place when $|{{{\mathrm{P}}}}_{{{{\mathrm{abs}}}}}^{{{{\mathrm{eff}}}}}({{{\mathrm{t}}}} - {{{\mathrm{t}}}}_0)|\, > \,{{{\mathrm{P}}}}_{{{{\mathrm{TH}}}}}$, which can be simplified to ${{{\mathrm{P}}}}_{{{{\mathrm{abs}}}}}({{{\mathrm{t}}}} - {{{\mathrm{t}}}}_0)\, > \,{{{\mathrm{P}}}}_{{{{\mathrm{TH}}}}}$ considering that the leaked power ${{{\mathrm{P}}}}_{{{{\mathrm{leak}}}}}$ is negligible (see Fig. S10 in the Supplementary Material). In the post-sliding period, the absorbed optical power rapidly decreases. Concurrently, the slow cooling process begins with $|{{{\mathrm{P}}}}_{{{{\mathrm{abs}}}}}^{{{{\mathrm{eff}}}}}(t - t_0)|\, < \,{{{\mathrm{P}}}}_{{{{\mathrm{TH}}}}}$. Accordingly, the contact surface ceases the sliding (see Fig. 1c), while preserving the previously accumulated sliding distance thanks to the static friction force that prevents the contact surface from returning to its original position. Thus, in the whole process, the friction force plays a double role: mitigating the actuation in the sliding period, while preserving the actuation in the post-sliding period. Moreover, as shown in Fig. 1d, the stabilized sliding distance decreases as the sliding resistance ${{{\mathrm{F}}}}_{{{{\mathrm{slide}}}}}$ increases. Notably, the sliding completely ceases when ${{{\mathrm{F}}}}_{{{{\mathrm{slide}}}}}$ exceeds a critical value (~35 µN), where ${{{\mathrm{P}}}}_{{{{\mathrm{TH}}}}}$ approximately equals the absorbed peak power.

### Spiral motion of gold plates

**Fig. 2:**
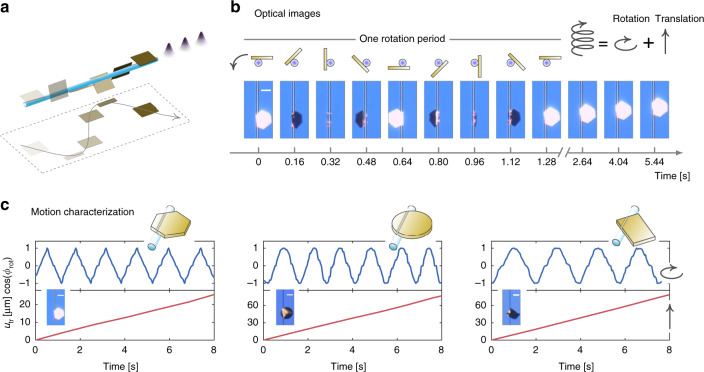
Systematic measurement of spiral motion of gold plates around micro-fibers in ambient conditions. **a** Sketch of the observed spiral motion. **b** Temporal sequencing of optical images of a hexagonal gold plate spirally moving around a micro-fiber. The fiber has a diameter of 2 µm, and the side length and the thickness of the plate are 27.72 µm and 30 nm, respectively. **c** Cosine of rotation angle *Φ*_rot_ (upper panels), translation displacement (lower panels) as functions of time for gold plates with hexagonal, circular and rectangular base shapes. All scale bars represent 15 µm. The used super-continuum laser pulses have 6.8-mW average power, 2.6-ns temporal width and 6.13–kHz repetition rate

Experimentally, in ambient conditions, we set up a gold-plate and micro-fiber coupled system (Fig. 2a)—which hosts two-dimensional locomotion of gold plates on curved surfaces (that is, rotation and translation in the azimuthal and axial directions of the micro-fibers, respectively)—to concretize the proposed theoretical concept. We fabricated gold plates of various base shapes, including triangle, rectangle, square, hexagon, and circle (see Fig. S14 in the Supplementary Material), to evidence that the proposed scheme works insensitive of structure geometry. The plates have thicknesses varying between 30 nm and 60 nm, and their base dimensions are typically about a few tens of micrometers. The micro-fibers with a diameter about a few micro-meters (1.5–4 µm) were tapered from standard optical fibers using the flame-heated drawing technique. The plates were placed on top of the micro-fibers. They stick together by the adhesive surface force of the order of µN, and accordingly, support friction force of the same order ^21,23^.

To surpass the sliding threshold power endowed by the friction force, while mitigating undesirable heating effects, we used an ns-pulsed supercontinuum light source (wavelength range, 450 nm to 2400 nm; see Fig. S9A in the Supplementary Material for the laser spectrum) to drive the plates. As the laser pulses propagate inside the micro-fibers, their evanescent electric fields leak out and interact with the plates, which are then converted into heat by Ohmic losses of gold (see Fig. S9C for measured absorption spectrum in the Supplementary Material). The electromagnetic simulations (see Fig. S8 in the Supplementary Material) demonstrate that the fiber modes with a dominant electric field component perpendicular to the plate surface can be more efficiently absorbed by the gold plates due to plasmonic effects than those without^32^. Moreover, the absorbed optical power localizes within a narrow line-shaped subwavelength region centralized at the touching line between the plate and the micro-fiber as a result of plasmonic confinements.

The nanosecond pulsed optical absorption—spatially overlapping with the contact surface—excites the fundamental elastic *L*- and *T*-modes (together with other less important high-order modes). These modes bounce back and forth inside the plates, rendering the locomotion of the plates two degrees of freedom: rotation (*L*-modes) and translation (*T*-modes). As the elastic waves pass through the contact surface, they interact with the friction force following the elementary process revealed in the pedagogical model (Fig. 1), and drive the plates to slide. The combination of rotation and translation freedoms results in the spiral motion of the plates, as shown in Fig. 2b with the recorded temporal sequential optical images for a hexagonal plate. This observation generalizes the previously observed single motion freedom, either rotation^23^ or translation^22,24^. Specifically, the hexagonal plate translates towards the upper axial direction of the micro-fiber with a speed of 3.4 µm/s, and, at the meantime, it rotates anti-clockwise (as viewed from the top side of the optical images) with a speed of 46 rpm (characterized in the leftmost panel of Fig. 2c). The measured translation and rotation speeds increase approximately linearly with the repetition rate of the laser pulses (see Fig. S17 in the Supplementary Material and Supplementary Movie 1), evidencing that the spiral motion of the plates is driven by individual single pulses. Besides, the spiral motion was observed for gold plates with various geometries (see Fig. 2c, Fig. S15 in the Supplementary Material, and Supplementary Movie 2), and with the side length up to two hundred micrometers. The gold plates can stably move on the micro-fibers over a distance of several centimeters. We also performed experiments in a vacuum chamber with the same observations on the spiral motion of the gold plates (see Fig. S19 in the Supplementary Material), which thus indicates that air molecules here play a negligible role.

### Motion control

**Fig. 3:**
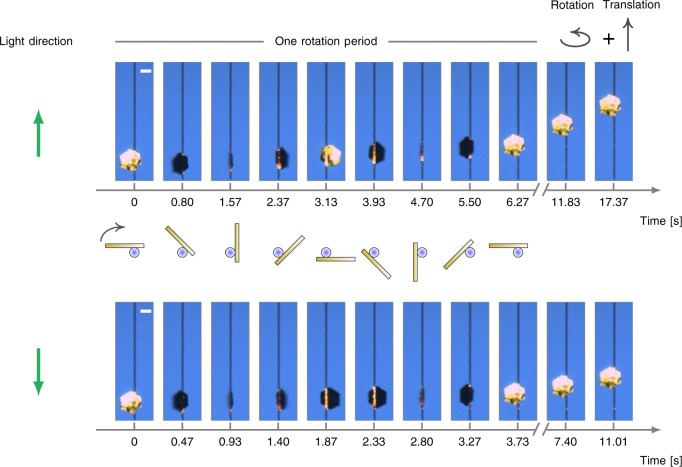
Sequential optical images of a hexagonal gold plates moving around micro-fibers spirally in ambient conditions, showing that both rotation and translation directions are independent of incident light directions. All scale bars represent 15 µm. The laser source has the same parameters as in Fig. 2

As an essential step towards realizing practical actuators, a complete control of motion direction is indispensable. For actuators driven by optical force, their control could be implemented by changing the propagation directions of the incident light. However, the same scheme fails here, wherein the dominant force is the friction force. Instead, we observed that the motion directions of the gold plates are independent of the light directions, as shown in Fig. 3 and Supplementary Movie 3. This observation has been confirmed with similar tests involving 40 different gold plates^34^. Below, we shall demonstrate that both rotation and translation directions can be manipulated by adjusting how the gold plates are placed on and in contact with the micro-fibers.

**Fig. 4:**
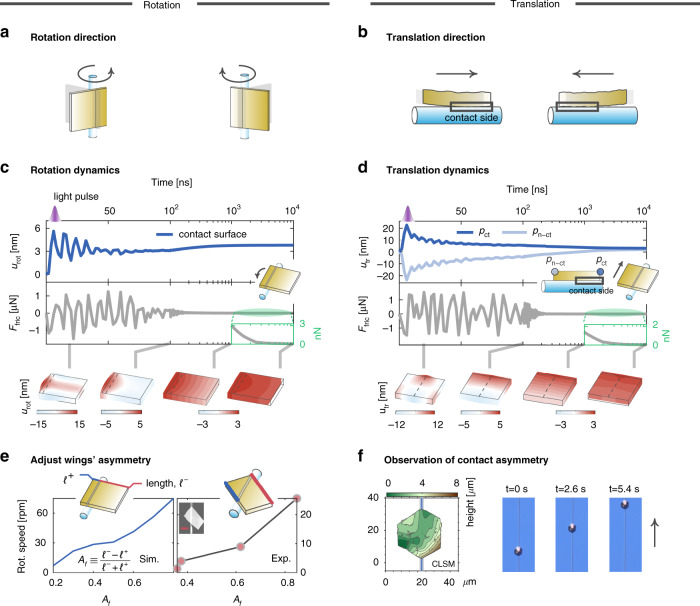
Mechanism of spiral motion of gold plates. The spiral motion consists of two degrees of freedom—rotation (**a**, **c**, **e**) and translation (**b**, **d**, **f**). **a**, **b**. Sketch of rotation (**a**) and translation (**b**) directions of gold plates and of their dependencies on wings’ and contact-surface’s asymmetries, respectively. **c**, **d** Simulation results of (1) sliding displacements (top panels) and (2) friction forces (middle panels) as functions of time for square gold plates driven by a single laser pulse, and of (3) displacement profiles at different time (lower panels). The square plates have 30-nm thickness and 10-µm side length; the frictional sliding resistance is 1.5 µN; the life time of the elastic waves is 20 ns; the fiber has a diameter of 1.8 µm. The pulsed optical absorption has a 100-mW peak power and a 3-ns width, and its spatial distribution is specified in Eq. (5.1) in the Supplementary Material. Moreover, in the rotation (**c**) case, the two wings of the plate have unequal lengths, 8 µm and 2 µm, wherein the translation freedom is locked due to the absence of the contact asymmetry. In the translation (**d**) case, the two wings are of equal length to lock the rotation freedom, and the contact surface situates in the half side of the plate. The temperature distributions at different time in the plate are provided in Figs. S11B and S12B in the Supplementary Material. **e** Simulated (left) and measured (right) rotation speeds as functions of wings’ asymmetry factor *A*_*f*_. The repetition rate of laser pulses is 2.04-kHz. Scale bar: 4 µm. **f** Confocal laser scanning microscopy image (left) and temporal sequencing of optical images (right) of a hexagonal gold plate on a micro-fiber, evidencing the direct correlation between the contact asymmetry and the translation direction

*Rotation*.—The dimension asymmetry in two wings of the gold plates—demarcated by the micro-fibers—is the key to enabling the rotation of the plates (Fig. 4a), which has been firstly observed by Lu et al.^23^, and is confirmed here for the spiral motion. As shown in Fig. 2b and Fig. S15 in the Supplementary Material, the rotation direction points from the long wing to the short wing (that is, the long wing seems to push forward the short wing to rotate). In the presence of the symmetry in two wings, the rotation stops. The simulation and experimental results in Fig. 4e confirm this, showing that, the rotation speed monotonically decreases as the asymmetry factor $A_f$ (see the inset for the definition of $A_f$) decreases (i.e., two wings become more symmetric).

The rotation mechanism can be qualitatively comprehended with the insights from the pedagogically model of Fig. 1, and by additionally taking multiple reflections of the *L*-modes due to finite size effects of the gold plates into account. For a more precise quantitative prediction, one should refer to numerical simulations as will be discussed with Fig. 4. Briefly, the optical-absorption hotspot centralized around the contact surface generates the *L*- modes, which propagate towards the long- and short-wing sides, and, for convenience, are termed as “long-wing (LW)” and “short-wing (SW)” waves, respectively. These initial LW and SW waves carry the dominant elastic displacements towards the long-wing and short-wing sides, respectively. As these LW and SW waves reach the ends of the plates, their propagation directions reverse and turn towards the contact surface. Next, when these waves return back to the contact surface, the contact surface rotates as a result of the elementary interaction between the incident elastic waves and the friction force (see Fig. 1). This elementary interaction continuously happens as the *L*-modes bounce back and forth inside the plates, manifesting in the temporal oscillations of the rotation displacement (see the upper panel in Fig. 4c); and the sliding gradually weakens due to elastic attenuation. This is indicated in Eq. (1) that the power threshold increases with the propagation time $t_0$ of the reflected elastic waves. Notably, albeit the reversion of the propagation direction due to the reflection, the LW and SW waves retain the same directions of the elastic oscillations, i.e., pointing towards the long- and short-wing sides, respectively. This is due to that the reflection adds negligible phase changes to the *L*-modes (see Fig. S7 in the Supplementary Material). Consequently, the LW and SW waves consistently drive the plates to rotate towards the long- and short-wing sides oppositely. The net rotation points towards the short-wing side since the SW waves are less attenuated thanks to their shorter traveling distance. This process is captured with the simulated profiles of the elastic displacement in the rotation direction at different time, as shown in the lower panel of Fig. 4c.

In this process, the friction force plays the same double role as in Fig. 1c. First, as the contact surface initially rotates towards the short-wing side, the friction force on average points towards the long-wing side resisting the rotation (see the middle panel in Fig. 4c). Then, as time increases and the thermal cooling begins, the friction force plays an opposite role: it instead points towards the short-wing side against thermal contraction (see the inset in the middle panel of Fig. 4c), thereby preventing the contact surface from returning to its original position. The finishing of this cooling process takes a time of about 10^4^ ns (see Fig. S10B in the Supplementary Material).

*Translation*.—The translation of the gold plates essentially requires the breaking of reflection symmetry in the axial direction of the micro-fibers. Otherwise, if the reflection symmetry is respected, there is no directional bias to trigger the translation. Here, the asymmetry in the contact surface is identified to be the key enabling the translation, which is consistent with our observation that the translation direction is independent of light direction. This novel translation mechanism marks the essential difference between our scheme and another in a recent publication^24^—that proposes the asymmetry in the spatial distribution of optical absorption as the key factor and observes the dependence of the translation direction on light direction (see Sec. 6-B2 in the Supplementary Material for more discussions).

The origin of the contact asymmetry is the surface curvature of the plates. Interestingly, the surface curvature is unintentionally introduced during the process of transferring the plates onto the micro-fibers. The plates are initially placed on glass substrates. In order to peal the plates off the substrates and then drag them to the micro-fibers, the tapered fibers are used as levers to tilt the plates up. The plates are inevitably bent (see Fig. S20 in the Supplementary Material). Notably, the surface curvature can even be as large as a few micro-meters, as shown by confocal laser scanning microscopy (CLSM) images in Fig. 4f and Fig. S21 in the Supplementary Material.

As a result of the existing surface curvature, the real contact surface potentially only occupies parts of the ideal flat surface (in the absence of the surface curvature), thereby dividing the latter into the non-contact and contact sides, respectively (see Fig. 4b and f). The temporal sequencing of optical images for a hexagonal plate in Fig. 4f shows that the translation direction points from the non-contact side to the contact side. We numerically reproduce the same observation in Fig. 4d. Specifically, the upper panel of Fig. 4d contrasts the temporal evolution of the translation displacements of two points on the non-contact (*P*_n−ct_) and contact (*P*_ct_) sides, respectively, and shows that the stabilized translation direction points from the *P*_n−ct_ point to the *P*_ct_ point.

The dependence of the translation direction on the contact asymmetry can be appreciated by analyzing the friction force (see the middle panel in Fig. 4d). First, in the initial sliding period, the thermally excited elastic waves expand the two ends of the plate in opposite directions, and the friction force on average pointing towards the *P*_n−ct_ point resists the thermal expansion of the contact side. Next, as the thermal cooling starts, the friction force instead, pointing towards the *P*_ct_ point, preserves the previously accumulated sliding distance of the contact surface, and accordingly, drags the non-contact side toward the contact side (see the lower panel in Fig. 4d). Consequently, the net motion direction is towards the contact side, which physically relates with the excitations of the *T*- modes whose elastic oscillation direction is parallel to the axial direction of the micro-fiber (see the inset in Fig. 1b).

Note that, in the cooling period, the contact side does not stay in place after the initial quick expansion, and instead slowly reduces to a smaller value. This is attributed to that the excited elastic waves bounce back and forth inside the plate, resulting in a homogenized distribution of the elastic waves, and accordingly, making the translation displacements of the two sides approach each other. With these insights, we underline that the translation direction can be controlled by adjusting the contact asymmetry. In view of this, to adjust the translation direction, one can either rotate the plates with an angle of π or change the relative positions between the plates and the micro-fibers (see Fig. S18 in the Supplementary Material and Supplementary Movie 4), so that the spatial configurations of the non-contact and contact sides could change.

## Discussions and conclusion

The experimental results presented here demonstrate the two-dimensional locomotion of micro-scale objects on dry surfaces in the presence of µN-scale friction force by exploiting thermally excited elastic waves. The theoretical analysis—reinforced by the experimental results—reveals the elementary interaction dynamics between elastic waves and friction force, highlighting the double role of friction force in both hindering and promoting actuation at different stages. The feasibility of the proposed scheme manifests in mild mW-scale threshold optical absorption instantaneous power ${{{\mathrm{P}}}}_{{{{\mathrm{TH}}}}}$ required to enable actuation.

**Fig. 5:**
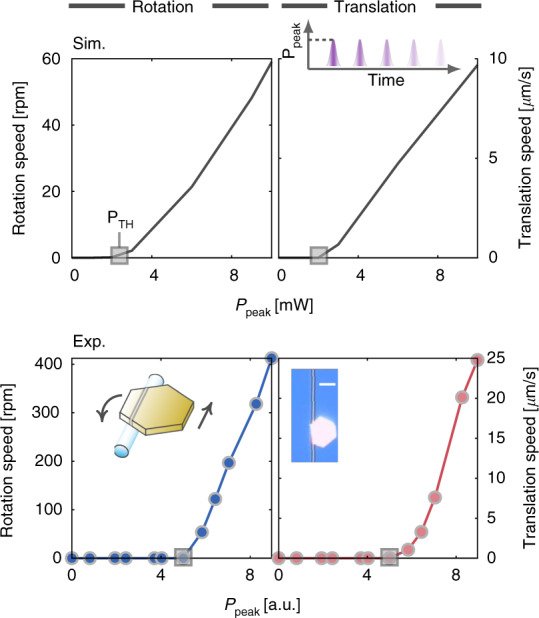
Observation of sliding threshold power PTH in spiral motion of gold plates. The numerical results of rotation and translation speeds as functions of absorbed peak power (top panel) for rectangular gold plates [same as those studied in Fig. 4c (rotation) and d (translation), respectively] feature the mW-scale sliding power threshold P_TH_, which qualitatively agree with the measurement results (lower panel) for a hexangular plate (same as in Fig. 2b). The numerical simulations adopt the same parameters of the pulsed absorbed optical power, the micro-fiber, and the frictional sliding resistance as in Fig. 4c–d. The experimentally used super-continuum laser pulses have 6.8-mW average power, 2.6-ns temporal width and 17.50-kHz repetition rate. The scale bar in the inset optical image is 15 µm

Experimentally, we observed the threshold power ${{{\mathrm{P}}}}_{{{{\mathrm{TH}}}}}$ by monitoring the power dependence of the rotation and translation speeds (see Fig. 5 and Supplementary Movie 5, noting the qualitative agreement between the theoretical and experimental results). This result—together with the predicted linear relation between ${{{\mathrm{P}}}}_{{{{\mathrm{TH}}}}}$ and friction force [Eq. (1)]—suggests that the proposed actuation system can also be used to probe, analyze friction force at micro-scales by monitoring changes of ${{{\mathrm{P}}}}_{{{{\mathrm{TH}}}}}$, and further investigate friction laws. Regarding this, a qualitative determination of the friction resistance requires a precise measurement of ${{{\mathrm{P}}}}_{{{{\mathrm{TH}}}}}$. This requirement favors the use of a narrow-band pulsed laser, with which ${{{\mathrm{P}}}}_{{{{\mathrm{TH}}}}}$ could be more precisely measured, e.g., using a power meter that generally has a limited bandwidth.

The demonstrated locomotion is driven by single laser pulses in a stepwise fashion. The step actuation distance can be as small as a few nanometers and even approach sub-nanometers. The motion speed increases with the pulse repetition rate and the pulse power. In addition, we discover that the motion direction is controllable by mechanically adjusting the relative positions and contact configurations between the gold plates and the microfibers. Further, to improve this controllability—particularly relating to the contact configurations––one practical direction is to fabricate micro-plates with predefined surface curvatures, e.g., with electron-beam lithography methods. Besides, it is also meaningful to explore new designs for the realization of the motion control in a full-optical way, i.e., by tuning the propagation direction, wavelength, and fiber modes of the incident laser pulses (see Sec. 6-B2 in the Supplementary Material).

We envision the proposed actuation scheme can in principle find practical applications in various fields that require precisely manipulate micro-objects in non-liquid environments. For instance, integrating our technique with an on-chip waveguide coupled network^36^, one can in principle achieve optical modulation by adjusting positions of a gold plate on top of the waveguide to control waveguide transmission via tuning coupling between nearby waveguides. Moreover, it can also be used for transporting dielectric particles attached to the surface of a gold plate along a micro-fiber/nano-wire, which is essential in lab-on-a-chip technologies, e.g., for life-science applications.

## Materials and methods

### Experiments

The micrometer-sized hexagonal gold plates are synthesized in large quantities by introducing aniline (C_6_H_7_N) to a heated ethylene glycol (EG) solution of hydrogen tetrachloroaurate (HAuCl_4_·4H_2_O), following the recipe reported in Ref ^35^. The typical side length and thickness of synthesized gold plates are in the order of several tens of micrometers and of nanometers, respectively. The gold plates in the shape of triangle, circle, rectangle, and square are fabricated by electron beam lithography. The process was carried out on a SiO_2_ substrate spin-coated at 5000 r.p.m. with poly (methyl methacrylate) (MicroChem 950 PMMA A4) electronic resist. An electron beam lithography machine (ELPHY Quantum, Raith, Germany) equipped with a pattern generator was used for direct writing the designed micro-structures. Then the exposed resist was developed in a conventional solution of methyl isobutyl ketone-isopropyl alcohol (MIBK-IPA) (1:3) for 60 s. Using electron beam evaporation, 60-nm Au was deposited on the sample. Finally, a gap was formed at the interface between the exposed and unexposed regime, making it easy to transfer towards the microfiber by a tapered fiber.

The microfibers are fabricated using a flame-heated drawing technique from standard optical fibers^37^. A hydrogen flame is used for heating the fiber. Then, the fiber is stretched and elongated gradually with the reduced diameter until the desired diameter of the microfiber is reached, under an optimized pulling force exerted on the standard fiber at both ends.

The used pulsed supercontinuum light (SuperK Compact, NKT photonics) has a wavelength range of 450–2400 nm, a pulse duration of 2.6 ns, and tunable repetition rates ranging from 230 Hz to 23 kHz. The variable optical attenuator is used for controlling the output attenuation. Then, the pulsed light with tunable single pules energy is first lens-coupled into a standard silica fiber (SMF-28, Corning) and next delivered into a microfiber. The movements of the gold plates on microfibers in ambient conditions are monitored using a 10X microscope objective and a CCD camera.

### Simulations

Numerical simulations, in Fig. 1c and d, Fig. 4c and d, are performed using the commercial finite-element simulation software, COMSOL Multiphysics. The coupled heat-mechanical simulations are performed by coupling the COMSOL modules of “Heat Transfer in Solids” and “Solid Mechanics”. The friction force is introduced through the augmented Lagrangian method using the contact node under the “Solid Mechanics” module. The COMSOL files can be requested by contacting WY.

## Supplementary information

SI—Micro-scale opto-thermo-mechanical actuation in the dry adhesive regime

Supplementary Video 1

Supplementary Video 2

Supplementary Video 3

Supplementary Video 4

Supplementary Video 5
