# Supplementary material for: Micro-scale opto-thermo-mechanical actuation in the dry adhesive regime
Source: Light Sci Appl. 2021 Sep 22;10:193. doi: 10.1038/s41377-021-00622-6 (PMC8458461; doi:10.1038/s41377-021-00622-6)
Supplement: Supplementary file 1 — SI—Micro-scale opto-thermo-mechanical actuation in the dry adhesive regime [file 41377_2021_622_MOESM1_ESM.pdf]

SUPPLEMENTARY INFORMATION

Micro-scale opto-thermo-mechanical actuation in the dry adhesive regime

Weiwei Tang,[1,2] Wei Lv,[1,2] Jinsheng Lu,[3] Fengjiang Liu,[1,2] Jiyong Wang,[1,2] Wei Yan,[1,2] and Min Qiu[1,2]

[1]*Key Laboratory of 3D Micro/Nano Fabrication and Characterization of Zhejiang Province, School of Engineering, Westlake University, 18 Shilongshan Road, Hangzhou 310024, Zhejiang Province, China*

[2]*Institute of Advanced Technology, Westlake Institute for Advanced Study, 18 Shilongshan Road, Hangzhou 310024, Zhejiang Province, China*

[3]*State Key Laboratory of Modern Optical Instrumentation, College of Optical Science and Engineering, Zhejiang University, Hangzhou 310027, China*

CONTENTS

1. Elastic waveguide modes in gold rectangular plates 1
   A. 2D Slab 2
   B. 3D rectangular plate 4

2. Elastic waves Excited by Temperature Change and Friction Force 5
   A. Modal Expansion of Elastic Green's Tensor 6
   B. Approximations 7
   C. Case Study: Fig.1 in the main text 7

3. Rotation Displacement of Gold Plates around Micro-fibers: Theoretical Analysis 12

4. Optical Absorption in Gold-Plate & Micro-fiber Coupled System 14

5. Heating and Cooling Dynamics in Gold-Plate & Micro-fiber Coupled System 15

6. Coupled Heat-Elastic Simulations 17
   A. Rotation 17
   B. Translation 19

7. Fabricated gold plates 20

8. Experimental characterizations of spiral motion of gold plates around micro-fibers 21
   A. Optical Images of Spiral Motion 21
   B. Controlling Motion Speed by Varying Repetition Rate of Laser Pulses 24
   C. Manipulating Spiral Motion by Adjusting Relative Positions between Gold Plates and Micro-fibers 25
   D. Spiral Motion in a Vacuum Chamber 25

9. Surface Topography of Gold Plates 25

References 26

1. ELASTIC WAVEGUIDE MODES IN GOLD RECTANGULAR PLATES

In this section, we derive dispersion relations of elastic waveguide modes in gold plates with rectangular cross section, **supplementing Fig. 1B in the main text**.

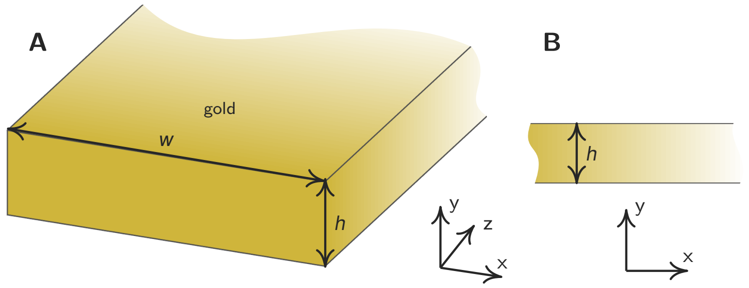

FIG. S1: **Sketch of elastic waveguides made of gold. A.** A three-dimensional gold plate with rectangular cross section. **B.** A two-dimensional gold slab. The elastic waveguide modes of the 2D slab are used as the basis to represent the modes of the 3D plate and to derive the dispersion relations of the latter.

As shown in Fig. S1A, the cross section (in the $x-y$ plane) of the studied gold plate has geometrical dimensions of width h and thickness h, and the plate extends to the infinity in the z-direction. Elastic

waveguide modes are eigensolutions of the frequency-domain linear elastic equation, read as [1]

$$\nabla \times \nabla \times \mathbf{u}(\mathbf{r};\omega) - \frac{2(1-\mu)}{1-2\mu}\nabla\nabla\cdot\mathbf{u}(\mathbf{r};\omega) - \omega^2\frac{2\rho(1+\mu)}{E}\mathbf{u}(\mathbf{r};\omega) = 0, \tag{1.1a}$$

and they are constrained by the boundary conditions (BCs)—surface traction vectors (force per unit area) on the plate's outside walls vanish—:

$$\hat{\mathbf{n}}\cdot\boldsymbol{\sigma}(\mathbf{r};\omega) = 0, \tag{1.1b}$$

where $\mathbf{u} \equiv u_x\hat{x} + u_y\hat{y} + u_z\hat{z}$ denotes the elastic displacement vector; ρ, E and μ denote the mass density, Young's modulus and Poison's ratio of gold, respectively; σ is the stress tensor.

Note that it is difficult to derive analytic expressions for waveguidng modes of 3D rectangular plates directly. Nevertheless, since the plates used in our experiments are very thin with h only about tens of nanometers, the long wavelength approximation along the thickness dimension (requiring $h \ll \lambda$, where λ denotes elastic wavelength of our interest) can be exploited to simplify derivations. Moreover, we will firstly start with a simpler case, a two-dimensional (2D) slab (Fig. S1B), and then use the modes of the slab (derived under the long wavelength approximation) as the basis to construct, derive the modes of 3D plates.

A. 2D Slab

A 2D slab, with thickness h extending from $-h/2$ to $h/2$ in the y-direction, is sketched in Fig. S1B. The elastic waveguide modes propagate in the x-direction and their wavenumbers are denoted by $\beta_{2\mathrm{D}}$. The displacement vectors of the modes, denoted by $\mathbf{u}_{2\mathrm{D}} \equiv u_{2\mathrm{D};x}\hat{x} + u_{2\mathrm{D};y}\hat{y} + u_{2\mathrm{D};z}\hat{z}$, are represented by linear combinations of transverse and longitudinal elastic plane waves in bulk gold:

$$u_{2\mathrm{D};x} = [T_1 k_y^{\mathrm{T}}\cos\left(k_y^{\mathrm{T}}y\right) + T_2 k_y^{\mathrm{T}}\sin\left(k_y^{\mathrm{T}}y\right) + L_1\beta\cos\left(k_y^{\mathrm{L}}y\right) + L_2\beta k_y^{\mathrm{L}}\sin\left(k_y^{\mathrm{L}}y\right)]\exp(i\beta_{2\mathrm{D}}x), \tag{1.2a}$$

$$u_{2\mathrm{D};y} = [-iT_1\beta\sin\left(k_y^{\mathrm{T}}y\right) + iT_2\beta\cos\left(k_y^{\mathrm{T}}y\right) + iL_1k_y^{\mathrm{L}}\sin\left(k_y^{\mathrm{L}}y\right) - iL_2k_y^{\mathrm{L}}\cos\left(k_y^{\mathrm{L}}y\right)]\exp(i\beta_{2\mathrm{D}}x), \tag{1.2b}$$

$$u_{2\mathrm{D};z} = [T_3 k_y^{\mathrm{T}}\cos\left(k_y^{\mathrm{T}}y\right) + T_4 k_y^{\mathrm{T}}\sin\left(k_y^{\mathrm{T}}y\right)]\exp(i\beta_{2\mathrm{D}}x), \tag{1.2c}$$

with

$$\underbrace{\left(k_y^{\mathrm{T}}\right)^2 + \beta_{2\mathrm{D}}^2 = \omega^2\frac{2\rho(1+\mu)}{E}}_{\text{dispersion relation of transverse plane waves}}, \quad \underbrace{\left(k_y^{\mathrm{L}}\right)^2 + \beta_{2\mathrm{D}}^2 = \omega^2\frac{\rho(1+\mu)(1-2\mu)}{E(1-\mu)}}_{\text{dispersion relation of longitudinal plane waves}}, \tag{1.2d}$$

where $T_{1,2,3,4}$ and $L_{1,2}$ are the (unknown) modal coefficients of the transverse and longitudinal plane waves, respectively.

We solve the dispersion relations of the elastic waveguide modes of the 2D slab (i.e., $\beta_{2\mathrm{D}}$ as a function of ω) by plugging Eqs. (1.2) into the BCs, Eq. (1.1b). Notably, as a result of the reflection symmetry of the slab about the x-axis, the waveguide modes can be partitioned into four sets, relating to the different terms in Eqs. (1.2) associated with $\{T_1, L_1\}$, $\{T_3\}$, $\{T_2, L_2\}$ and $\{T_4\}$, respectively. Among the four decoupled solutions, the former two deserve more attentions, because they lead to fundamental modes showing no cutoff and more importantly because their linear combinations give the essential modes of 3D plates (i.e., fundamental L- and T-like modes, highlighted in Fig. 1B in the main text) that are relevant to our experimental observations. Thereupon, we here below focus on the two sets of the modes concerning

$\{T_1, L_1\}$ and $\{T_3\}$, respectively. Employing the long wavelength approximation [1], we derive that:

$$\text{longitudinal-like slab modes:}\quad \beta_{2D}^{L} \simeq \omega\sqrt{\frac{\rho(1+\mu)(1-\mu)}{E}},$$

$$\mathbf{u}_{2D}^{L} \simeq Ne^{i\beta_{2D}^{L}x}\hat{x} - iN\frac{\beta_{slab}^{L}\mu}{1-\mu}ye^{i\beta_{2D}^{L}x}\hat{y}, \tag{1.3a}$$

$$\text{transverse slab modes:}\quad \beta_{2D}^{T} = \omega\sqrt{\frac{2\rho(1+\mu)}{E}},$$

$$\mathbf{u}_{2D}^{T} = Ne^{i\beta_{2D}^{T}x}\hat{z}. \tag{1.3b}$$

Here, the two modes are labeled by the superscripts "L" and "T", implying that their longitudinal (L) and transverse (T) characters, respectively. The longitudinal-like slab modes have dominant longitudinal component and negligible transverse component, while the transverse slab modes are the same as transverse plane waves in bulk gold.

We validate the predictive accuracy of Eqs. (1.3) by examining the dispersion relations of the waveguide modes of a gold slab with $h = 30$ nm. As shown in Fig. S2, the theoretical predictions (markers) from Eqs. (1.3) and the fully-numerical results (solid lines) obtained with the COMSOL Multiphysics show a remarkable agreement.

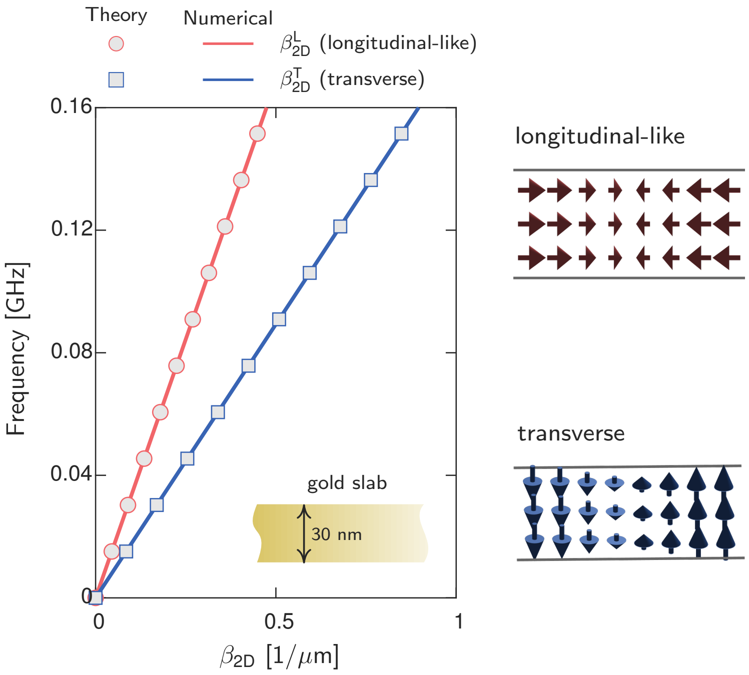

FIG. S2: **Validation of Eqs. (1.3) for predicting dispersion relations of fundamental elastic waveguide modes of a 2D gold slab with thickness 30 nm**. Right: modal profiles of longitudinal-like and transverse slab modes at the static frequency with arrows specifying oscillation directions of elastic waves.

---

[1] Approximate that $\cos\left(k_y^{T,L}y\right) \simeq 1$ and $\sin\left(k_y^{T,L}y\right) \simeq k_y^{T,L}y$.

B. 3D rectangular plate

We derive the dispersion relations of the waveguide modes in a 3D rectangular plate by expanding the displacement vector $\mathbf{u}_{3D}$ with $\mathbf{u}_{2D}^{L,T}$ derived in Eqs. (1.3):

$$u_{3D;x} = \left[T_1 \frac{\beta_{3D}}{\beta_{2D}^{T}} \sin\left(k_x^{T} x\right) - L_1 \frac{k_x^{L}}{\beta_{2D}^{L}} \sin\left(k_x^{L} x\right) - T_2 \frac{\beta_{3D}}{\beta_{2D}^{T}} \cos\left(k_x^{T} x\right) + L_2 \frac{k_x^{L}}{\beta_{2D}^{L}} \cos\left(k_x^{L} x\right)\right] \exp(i\beta_{3D} z), \tag{1.4a}$$

$$u_{3D;y} = \left[L_1 \frac{\mu}{1-\mu} \beta_{2D}^{L} y \cos\left(k_x^{L} x\right) + L_2 \frac{\mu}{1-\mu} \beta_{2D}^{L} y \sin\left(k_x^{L} x\right)\right] \exp(i\beta_{3D} z), \tag{1.4b}$$

$$u_{3D;z} = \left[iT_1 \frac{k_x^{T}}{\beta_{2D}^{T}} \cos\left(k_x^{T} x\right) + iL_1 \frac{\beta_{3D}}{\beta_{2D}^{L}} \cos\left(k_x^{L} x\right) + iT_2 \frac{k_x^{T}}{\beta_{2D}^{T}} \sin\left(k_x^{T} x\right) + iL_2 \frac{\beta_{3D}}{\beta_{2D}^{L}} \sin\left(k_x^{L} x\right)\right] \exp(i\beta_{3D} z), \tag{1.4c}$$

where β_{3D} denotes the wavenumber of a waveguide mode, and $\left(k_x^{L}\right)^2 + \beta_{3D}^2 = \left(\beta_{2D}^{L}\right)^2$ and $\left(k_x^{T}\right)^2 + \beta_{3D}^2 = \left(\beta_{2D}^{T}\right)^2$ [2].

In Eqs. (1.4), the waveguide modes can be sorted into two decoupled sets associated with $\{T_1, L_1\}$ and $\{T_2, L_2\}$, respectively, as a result of the reflection symmetry of the plate about the central $x-z$ plane. The two solutions correspond to the fundamental longitudinal (L)- and transverse (T)-like modes, respectively, which are highlighted in Fig. 1B in the main text. Employing the BCs, Eq. (1.1b), the approximated dispersion relations of the L- and T-modes are derived as

$$L\text{-modes:} \quad \left(\beta_{3D}^{L}\right)^2 \simeq \frac{\omega^2 \rho}{E} + \frac{\left(\beta_{3D}^{L}\right)^2 \left(k_x^{L}\right)^2 E}{\omega^2 \rho (\mu+1)^2} \left[\frac{\tan\left(k_x^{L} w/2\right) k_{\mathrm{x}}^{T}}{\tan(k_x^{T} w/2) k_{\mathrm{x}}^{L}} - 1\right], \tag{1.5a}$$

$$T\text{-modes:} \quad \left(\beta_{3D}^{T}\right)^2 \simeq \frac{\omega^2 \rho}{E} + \frac{\left(\beta_{3D}^{T}\right)^2 \left(k_{\mathrm{x}}^{L}\right)^2 E}{\omega^2 \rho (\mu+1)^2} \left[\frac{\cot\left(k_x^{L} w/2\right) k_{\mathrm{x}}^{T}}{\cot(k_x^{T} w/2) k_x^{L}} - 1\right]. \tag{1.5b}$$

Equations (1.5) belong to transcendental equations which blur closed-form solutions. In order to derive the closed-form solutions, we again take the long wavelength approximation (assuming $\beta_{3D}^{T,L} w \ll 1$) and obtain that

$$L\text{-modes:} \quad \beta_{3D}^{L} \simeq \omega \sqrt{\frac{\rho}{E}},$$
$$\mathbf{u}_{3D}^{L} \simeq N e^{i\beta_{3D}^{L} z} \left[-i\mu\beta_{3D}^{L} x\hat{x} - i\mu\beta_{3D}^{L} y\hat{y} + \hat{z}\right], \tag{1.6a}$$
$$T\text{-modes:} \quad \beta_{3D}^{T} \simeq \sqrt{\frac{\omega}{w}\left(\frac{12\rho}{E}\right)^{1/4}},$$
$$\mathbf{u}_{3D}^{T} \simeq N e^{i\beta_{3D}^{T} z} \left[\hat{x} + i\beta_{3D}^{T} x\hat{z}\right]. \tag{1.6b}$$

Note that the L-modes exhibit a linear dispersion under the long wavelength approximation (valid at low frequencies).

As shown in Fig. S3, we numerically validate the predictive accuracy of Eqs. (1.5) and (1.6) by examining the dispersion relations of the elastic waveguide modes in a gold rectangular plate with $h = 30$ nm, and $w = 10\,\mu$m (left panel), $5\,\mu$m (right panel). Here the theoretical results (markers) computed from Eqs. (1.5) agree excellently with the fully-numerical results obtained with the COMOSOL Multiphysics (solid lines), while the long-wavelength-approximation results (dashed lines) of Eqs. (1.6) are accurate for small frequencies.

---

[2] Note that the same notations $k_x^{L,T}$ are adopted in both Eqs. (1.4) and Eqs. (1.2), but with different meanings.

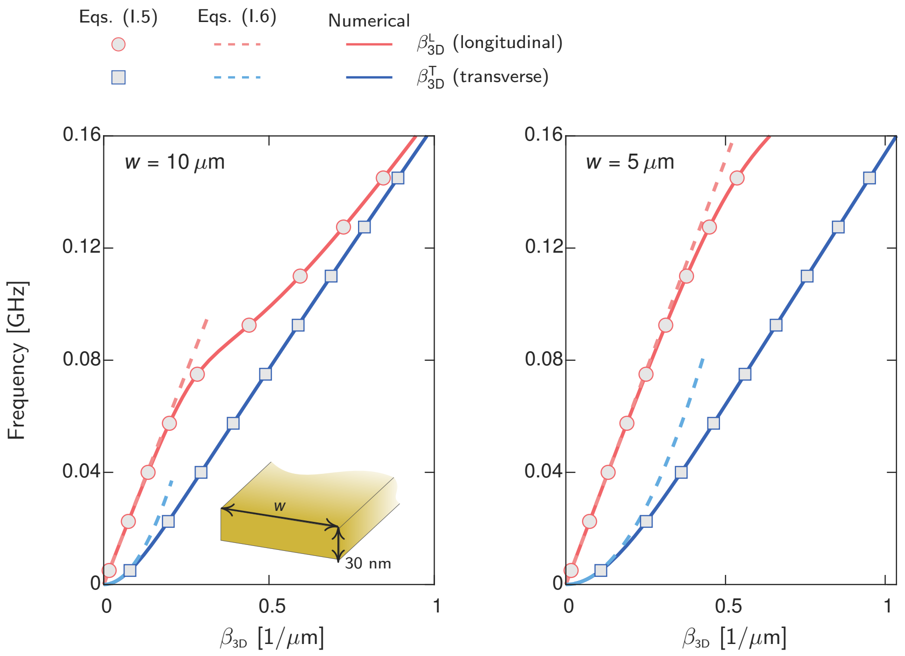

FIG. S3: **Validation of Eqs.** (1.5) **and** (1.6) **for predicting dispersion relations of elastic waveguide modes of gold rectangular plates**. The studied two plates have the same thickness, 30 nm, but different width, 10 μm (left) and 5 μm (right).

2. ELASTIC WAVES EXCITED BY TEMPERATURE CHANGE AND FRICTION FORCE

In this section, we derive the expressions of elastic displacements induced by temperature change (due to optical absorption) and friction force, **supplementing Figs. 1C-D and Eq. (1) in the main text**. For clarification, Fig. S4, duplicating Fig. 1A in the main text, sketches the investigated problem: locomotion of a rectangular gold plate on a curved support driven by pulsed optical absorption. Here, we focus on the elementary interaction between an incident (thermally-excited) elastic waves and reflected waves induced by the friction force, and, thus, omit the reflected elastic waves due to truncation of the plate in the z axis. For convenience of derivation, we set that the incident elastic wave is induced by a pulsed optical absorption that locates on the left side of the contact surface [3].

We start with the time-dependent linear elastic equation that relates the displacement vector of elastic waves with temperature change and friction force

$$\nabla \times \nabla \times \mathbf{u}(\mathbf{r};t) - \frac{2(1-\mu)}{1-2\mu}\nabla\nabla\cdot\mathbf{u}(\mathbf{r};t) + \frac{2\rho(1+\mu)}{E}\frac{\partial^2\mathbf{u}(\mathbf{r};t)}{\partial t^2} = -\alpha_{\text{th}}\frac{2(1+\mu)}{(1-2\mu)}\nabla\delta T(\mathbf{r};t) + \frac{2(1+\mu)}{E}\mathbf{f}_{\text{fric}}(\mathbf{r};t), \qquad (2.1)$$

where α_{th} denotes the coefficient of linear thermal expansion of gold, $\delta T \equiv T - T_0$ denotes the temperature change with respect to the ambient temperature $T_0 = 293$ K, and $\mathbf{f}_{\text{fric}}$ denotes the friction force density.

---

[3] This setting is different from our experiment, wherein the spatial distribution of the optical absorption overlaps with the contact surface, and generates elastic waves that bounce back and forth inside the plate. Nevertheless, the elementary physical process—interactions between an incident elastic force and friction force—is the same. Consequently, the results derived in this section can be applied to the experiment by taking multiple reflected elastic waves into account (see Sec. 3).

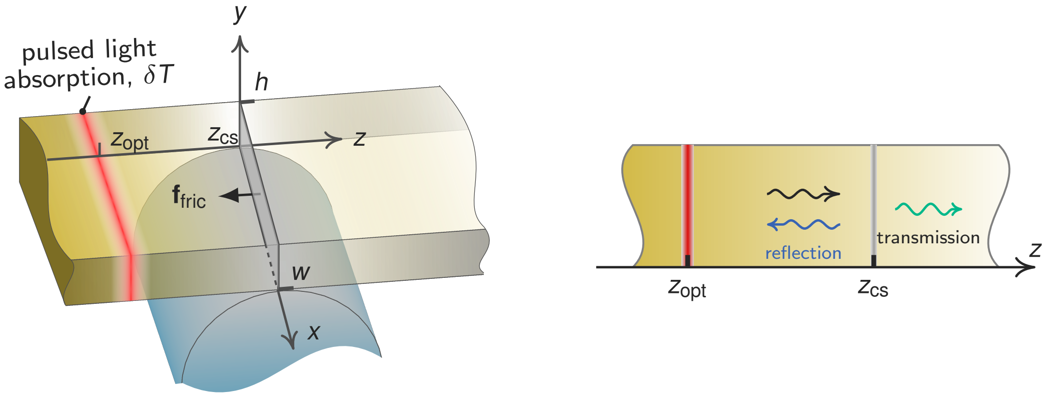

FIG. S4: **Sketch of the problem studied in Fig. 1 in the main text**. A gold plate contacts with a curved substrate. An incident light pulse is absorbed by the plate on the left side of the contact surface and induces a temperature change δT. The temperature change excites elastic modes, which, when passing through the line-shaped contact surface between the plate and the substrate, are resisted by friction force that induces reflected elastic waves. z_{op} and z_{cs} denote the centers of the z-coordinates of the optical absorbed power and the contact surface, respectively.

We express $\mathbf{u}$ in terms of δT and $\mathbf{f}_{fric}$ by

$$\mathbf{u}(\mathbf{r};t) = \int_{-\infty}^{t} \int d^3\mathbf{r}' \overline{\overline{\mathcal{G}}}(\mathbf{r},\mathbf{r}';t,t') \cdot \left[-\alpha_{th} \frac{2(1+\mu)}{(1-2\mu)} \nabla' \delta T(\mathbf{r}';t') + \frac{2(1+\mu)}{E} \mathbf{f}_{fric}(\mathbf{r}';t') \right], \tag{2.2a}$$

with elastic Green's tensor $\overline{\overline{\mathcal{G}}}(\mathbf{r},\mathbf{r}';t',t)$ defined by

$$\left[\nabla \times \nabla \times - \frac{2(1-\mu)}{1-2\mu} \nabla\nabla \cdot + \frac{\omega^2 \rho(1+\mu)}{E} \frac{\partial^2}{\partial t^2} \right] \overline{\overline{\mathcal{G}}}(\mathbf{r},\mathbf{r}';t,t') = \delta(\mathbf{r}-\mathbf{r}')\delta(t-t')\overline{\overline{\mathcal{I}}}. \tag{2.2b}$$

A. Modal Expansion of Elastic Green's Tensor

In this subsection, we represent $\overline{\overline{\mathcal{G}}}(\mathbf{r},\mathbf{r}';t,t')$ with the elastic waveguide modes of the plate. Firstly, we express $\overline{\overline{\mathcal{G}}}(\mathbf{r},\mathbf{r}';t,t')$ with the frequency-domain representation

$$\overline{\overline{\mathcal{G}}}(\mathbf{r},\mathbf{r}';t,t') = \frac{1}{2\pi} \int d\omega \overline{\overline{\mathcal{G}}}(\mathbf{r},\mathbf{r}';\omega) e^{-i\omega(t-t')}, \tag{2.3a}$$

where $\overline{\overline{\mathcal{G}}}(\mathbf{r},\mathbf{r}';\omega)$ defined by

$$\left[\nabla \times \nabla \times - \frac{2(1-\mu)}{1-2\mu} \nabla\nabla \cdot - \frac{2\omega^2 \rho(1+\mu)}{E} \right] \overline{\overline{\mathcal{G}}}(\mathbf{r},\mathbf{r}';\omega) = \delta(\mathbf{r}-\mathbf{r}')\overline{\overline{\mathcal{I}}}. \tag{2.3b}$$

Then, we denote the wavenumbers and the displacement vectors of the elastic waveguide modes by

$$\beta_{3D}^{\pm m} \quad \text{and} \quad \mathbf{u}_{3D}^{\pm m}(\mathbf{r};\omega) = \widetilde{\mathbf{u}}_{3D}^{\pm m}(x,y;\omega) e^{i\beta_{3D}^{\pm m} z},$$

where $\pm m = \pm 1, \pm 2, \cdots$ label modes propagating in the $\pm z$ directions, respectively. The $\pm m$ modes relate each other with

$$\beta_{3D}^{m} = -\beta_{3D}^{-m} \quad \text{and} \quad \widetilde{\mathbf{u}}_{3D}^{-m} = \widetilde{\mathbf{u}}_{3D}^{m} \cdot [-\hat{x}\hat{x} - \hat{y}\hat{y} + \hat{z}\hat{z}] \, .$$

The modal-expansion formulation of $\overline{\overline{\mathcal{G}}}(\mathbf{r},\mathbf{r}';\omega)$ is then given by

$$\overline{\overline{\mathcal{G}}}(\mathbf{r},\mathbf{r}';\omega)=\begin{cases}\sum_{m=1}^{\infty}\frac{i(v^{\mathrm{T}})^2}{2\omega v_{3\mathrm{D}}^m}\widetilde{\mathbf{u}}_{3\mathrm{D}}^{m}(x,y;\omega)\otimes\widetilde{\mathbf{u}}_{3\mathrm{D}}^{-m}(x',y';\omega)e^{i\beta_{3\mathrm{D}}^m|z-z'|} & \text{if } z>z' \\ \sum_{m=1}^{\infty}\frac{i(v^{\mathrm{T}})^2}{2\omega v_{3\mathrm{D}}^m}\widetilde{\mathbf{u}}_{3\mathrm{D}}^{-m}(x,y;\omega)\otimes\widetilde{\mathbf{u}}_{3\mathrm{D}}^{m}(x,y;\omega)e^{i\beta_{3\mathrm{D}}^m|z-z'|} & \text{if } z<z'\end{cases}, \tag{2.4}$$

where $v^{\mathrm{T}}=\sqrt{E/2\rho(1+\mu)}$ denotes the velocity of transverse plane waves in bulk gold, and $v_{3\mathrm{D}}^m=\partial\omega/\partial\beta_{3\mathrm{D}}^m$ is the group velocity for the mth waveguide mode. The modal vectors $\widetilde{\mathbf{u}}_{\mathrm{RWG}}^{\pm m}(x,y)$ are normalized such that

$$\int_{-w/2}^{w/2}dx\int_{-h/2}^{h/2}dy\,\widetilde{\mathbf{u}}_{3\mathrm{D}}^{-m}(x,y)\cdot\widetilde{\mathbf{u}}_{3\mathrm{D}}^{m}(x,y)=1.$$

B. Approximations

To derive a closed-form expression for $\overline{\overline{\mathcal{G}}}(\mathbf{r},\mathbf{r}';t,t')$ that benefits physics understanding, we propose the following approximations:

- only the L-modes are retained in the modal-expansion formulation of the Green's tensor,
- the long-wavelength approximate expressions for the L-modes, Eqs. (1.6), are used.

These approximations physically assume that the low-frequency L-modes are dominantly excited. This assumption is reasonable considering that: (i) we use an ns light pulse to drive the plate, so that the frequency of the excited elastic waves is below GHz, satisfying the low-frequency condition; (ii) the absorbed optical power is designed to distribute uniformly in the x-direction, so that the T-modes cannot be excited as a result of symmetry mismatching.

Taking Eqs. (1.6) into Eqs. (2.4) and then applying Eq. (2.3a), $\overline{\overline{\mathcal{G}}}(\mathbf{r},\mathbf{r}';t,t')$ is derived as

$$\overline{\overline{\mathcal{G}}}(\mathbf{r},\mathbf{r}';t,t')\simeq\begin{cases}\frac{(v^{\mathrm{T}})^2}{2Av_{3\mathrm{D}}^{\mathrm{L}}}\widetilde{\mathbf{u}}_{3\mathrm{D}}^{+\mathrm{L}}(x,y)\otimes\widetilde{\mathbf{u}}_{3\mathrm{D}}^{-\mathrm{L}}(x',y')H\left(t-t'-\frac{|z-z'|}{v_{3\mathrm{D}}^{\mathrm{L}}}\right) & \text{for } z>z' \\ \frac{(v^{\mathrm{T}})^2}{2Av_{3\mathrm{D}}^{\mathrm{L}}}\widetilde{\mathbf{u}}_{3\mathrm{D}}^{-\mathrm{L}}(x,y)\otimes\widetilde{\mathbf{u}}_{3\mathrm{D}}^{+\mathrm{L}}(x',y')H\left(t-t'-\frac{|z-z'|}{v_{3\mathrm{D}}^{\mathrm{L}}}\right) & \text{for } z<z'\end{cases}, \tag{2.5}$$

with

$$\widetilde{\mathbf{u}}_{3\mathrm{D}}^{\pm\mathrm{L}}(x,y)\equiv\pm\frac{\mu x\partial}{v_{3\mathrm{D}}^{\mathrm{L}}\partial t}\hat{x}\pm\frac{\mu y\partial}{v_{3\mathrm{D}}^{\mathrm{L}}\partial t}\hat{y}+\hat{z}, \tag{2.6}$$

where $H(x)$ denotes the Heaviside step function with $H(x)=1$ for $x\geq 0$ and $H(x)=0$ otherwise; $v_{3\mathrm{D}}^{\mathrm{L}}=\sqrt{E/\rho}$ denotes the velocity of the L-modes under the long-wavelength approximation; $A\equiv w\times h$ is the area of the cross section of the plate.

C. Case Study: Fig.1 in the main text

In this subsection, we employ Eqs. (2.5) and (2.6) to study Fig. 1 in the main text. We decompose the induced displacement vector $\mathbf{u}(\mathbf{r};t)$ into

$$\mathbf{u}(\mathbf{r};t)\equiv\mathbf{u}_{\mathrm{th}}(\mathbf{r};t)+\mathbf{u}_{\mathrm{fric}}(\mathbf{r};t), \tag{2.7}$$

where $\mathbf{u}_{\mathrm{th}}$ and $\mathbf{u}_{\mathrm{fric}}$ represent the contributions from the temperature change and the friction force, respectively.

1. Thermal contribution

Among three Cartesian components of $\mathbf{u}_{\mathrm{th}}$, its z component (parallel to the propagation direction of the L-modes), denoted by $u_{\mathrm{th};z}$, is dominant (see the modal profile in the inset of Fig. 1B in the main text).

$u_{\mathrm{th};z}$ is derived with the following algebra manipulations:

$$
\begin{aligned}
u_{\mathrm{th};z}(\mathbf{r};t) &= -\frac{2\alpha_{\mathrm{th}}(1+\mu)}{1-2\mu}\int_{-\infty}^{t}dt'\int d^3\mathbf{r}'\hat{z}\cdot\overline{\overline{\mathcal{G}}}(\mathbf{r},\mathbf{r}';t,t')\cdot\nabla'\delta T(\mathbf{r}';t') \\
&\overset{a}{\simeq} -\frac{\alpha_{\mathrm{th}}(v^{\mathrm{T}})^2(1+\mu)}{Av_{3\mathrm{D}}^{\mathrm{L}}(1-2\mu)}\int_{-\infty}^{t}dt'\int d^3\mathbf{r}'\left[\mathrm{sgn}(z'-z)\left(\frac{\mu x'\partial}{v_{3\mathrm{D}}^{\mathrm{L}}\partial t}\hat{x}+\frac{\mu y'\partial}{v_{3\mathrm{D}}^{\mathrm{L}}\partial t}\hat{y}\right)+\hat{z}\right]H\left(t-t'-\frac{|z-z'|}{v_{3\mathrm{D}}^{\mathrm{L}}}\right)\cdot\nabla'\delta T(\mathbf{r}';t') \\
&\overset{b}{=} \frac{\alpha_{\mathrm{th}}(v^{\mathrm{T}})^2(1+\mu)}{Av_{3\mathrm{D}}^{\mathrm{L}}(1-2\mu)}\int_{-\infty}^{t}dt'\int d^3\mathbf{r}'\,\delta T(\mathbf{r}';t')\nabla'\cdot\left\{\left[\mathrm{sgn}(z'-z)\left(\frac{\mu x'\partial}{v_{3\mathrm{D}}^{\mathrm{L}}\partial t}\hat{x}+\frac{\mu y'\partial}{v_{3\mathrm{D}}^{\mathrm{L}}\partial t}\hat{y}\right)+\hat{z}\right]H\left(t-t'-\frac{|z-z'|}{v_{3\mathrm{D}}^{\mathrm{L}}}\right)\right\} \\
&= \frac{\alpha_{\mathrm{th}}E}{2A\rho(v_{3\mathrm{D}}^{\mathrm{L}})^2}\int d^3\mathbf{r}'\,\mathrm{sgn}(z-z')\delta T\left(\mathbf{r}';t-\frac{|z-z'|}{v_{3\mathrm{D}}^{\mathrm{L}}}\right) \\
&\overset{c}{\simeq} \mathrm{sgn}(z-z_{\mathrm{opt}})\frac{\alpha_{\mathrm{th}}}{2A}\int d^3\mathbf{r}'\delta T\left(\mathbf{r}';t-\frac{|z-z_{\mathrm{opt}}|}{v_{3\mathrm{D}}^{\mathrm{L}}}\right).
\end{aligned}
\tag{2.8}
$$

The above derivations involve the following steps:

a. (1) Use $\overline{\overline{\mathcal{G}}}$ expressed in Eq. (2.5); (2) define the sign function $\mathrm{sgn}(x)$ such that $\mathrm{sgn}(x)=1$ for $x>0$, $\mathrm{sgn}(x)=-1$ for $x<0$ and $\mathrm{sgn}(x)=0$ for $x=0$.

b. Employ the technique of integration by parts.

c. Assume that δT localizes in a tiny region centralized at $z=z_{\mathrm{opt}}$ and approximate that $\frac{|z-z'|}{v_{3\mathrm{D}}^{\mathrm{L}}}\simeq\frac{|z-z_{\mathrm{opt}}|}{v_{3\mathrm{D}}^{\mathrm{L}}}$.

Departing from Eq. (2.8), we further establish a link between $u_{\mathrm{th};z}$ and the net heat energy absorbed into/leaked from the plate using the heat conduction equation,

$$
\rho c_p\frac{\partial\,\delta T(\mathbf{r};t)}{\partial t}-\nabla\cdot K\nabla\delta T(\mathbf{r};t)=Q_{\mathrm{abs}}(\mathbf{r};t),
\tag{2.9}
$$

where c_p and K denote the specific heat capacity and thermal conductivity of gold; Q_{abs} denotes the density of the absorbed optical power. With Eq. (2.9), we derive that

$$
\begin{aligned}
\int d^3\mathbf{r}\delta T(\mathbf{r};t) &\overset{a}{=} \int_{-\infty}^{t}dt'\int d^3\mathbf{r}Q_{\mathrm{abs}}(\mathbf{r};t')/(\rho c_p)+\int_{0}^{t}dt'\oint ds\hat{\mathbf{n}}\cdot K\nabla\delta T(\mathbf{r};t')/(\rho c_p) \\
&\overset{b}{=} \int_{-\infty}^{t}dt'\left[P_{\mathrm{abs}}(t')-P_{\mathrm{leak}}(t')\right]/(\rho c_p) \\
&\overset{c}{\equiv} W_{\mathrm{abs}}^{\mathrm{eff}}(t)/(\rho c_p).
\end{aligned}
\tag{2.10}
$$

The above derivations involve the following steps:

a. Apply the integral operation $\int_0^t dt'\int d^3\mathbf{r}$ to the both sides of Eq. (2.9) and employ the divergence theorem to transform the volume integral to the surface one.

b. Define the heat power leaked into the outside environment, $P_{\mathrm{leak}}(t)\equiv-\oint ds\hat{\mathbf{n}}\cdot K\nabla\delta T(\mathbf{r};t)$.

c. Define the effective heat energy with

$$
W_{\mathrm{abs}}^{\mathrm{eff}}\equiv\int_{-\infty}^{t}dt'\left[P_{\mathrm{abs}}(t')-P_{\mathrm{leak}}(t')\right].
$$

Inserting Eq. (2.10) into Eq. (2.8), we derive that

$$
u_{\mathrm{th};z}(\mathbf{r};t)\simeq\mathrm{sgn}(z-z_{\mathrm{opt}})\frac{\alpha_{\mathrm{th}}}{2A\rho c_p}W_{\mathrm{abs}}^{\mathrm{eff}}\left(t-\frac{|z-z_{\mathrm{opt}}|}{v_{3\mathrm{D}}^{\mathrm{L}}}\right).
\tag{2.11}
$$

2. Frictional contribution

Similar as $\mathbf{u}_{\text{th}}$, the z-component of $\mathbf{u}_{\text{fric}}$, denoted by $u_{\text{fric};z}$, is the dominant one, and is derived as follows

$$\begin{aligned} u_{\text{fric};z}(\mathbf{r};t) &= \frac{2(1+\mu)}{E}\int_{-\infty}^{t} dt' \int d^3\mathbf{r}'\hat{z}\cdot\overline{\overline{\mathcal{G}}}(\mathbf{r},\mathbf{r}';t,t')\cdot\mathbf{f}_{\text{fric}}(\mathbf{r}') \\ &\overset{a}{\simeq} \frac{2(1+\mu)}{E}\int_{-\infty}^{t} dt' \int d^3\mathbf{r}'\hat{z}\cdot\overline{\overline{\mathcal{G}}}(\mathbf{r},\mathbf{r}';t,t')\cdot\hat{z}f_{\text{fric};z}(\mathbf{r}') \\ &\overset{b}{\simeq} \frac{(v^{\text{T}})^2(1+\mu)}{Av_{\text{3D}}^{\text{L}}E}\int_{-\infty}^{t} dt' \int d^3\mathbf{r}' H\left(t-t'-\frac{|z-z'|}{v_{\text{3D}}^{\text{L}}}\right) f_{\text{fric};z}(\mathbf{r}';t') \\ &\overset{c}{\simeq} \frac{1}{2A\rho v_{\text{3D}}^{\text{L}}}\int_{-\infty}^{t} dt' H\left(t-t'-\frac{|z-z_{\text{cs}}|}{v_{\text{3D}}^{\text{L}}}\right) F_{\text{fric};z}(t'). \end{aligned} \tag{2.12}$$

The above derivations involve the following steps:

a. Application of $\mathbf{f}_{\text{firc}} \simeq f_{\text{firc};z}\hat{z}$.

b. Use $\overline{\overline{\mathcal{G}}}$ expressed in Eq. (2.5).

c. Note that the friction force exists on the contact surface between the plate and the substrate, which is a line-shaped area centralized at $z = z_{\text{cs}}$, so that $\int d^3\mathbf{r}' H\left(t-t'-\frac{|z-z'|}{v_{\text{3D}}^{\text{L}}}\right) f_{\text{fric};z}(\mathbf{r}';t') \simeq H\left(t-t'-\frac{|z-z_{\text{cs}}|}{v_{\text{3D}}^{\text{L}}}\right)\int d^3\mathbf{r}' f_{\text{fric};z}(\mathbf{r}';t') \equiv H\left(t-t'-\frac{|z-z_{\text{cs}}|}{v_{\text{3D}}^{\text{L}}}\right) F_{\text{fric};z}(t)$ with $F_{\text{fric};z}(t) \equiv \int d^3\mathbf{r} f_{\text{fric};z}(\mathbf{r};t)$.

3. Sliding Displacement & Threshold Power

We now employ Eqs. (2.11) and (2.12) to derive the expression for the sliding displacement of the contact surface, denoted by u_z^{cs}. By putting $z = z_{\text{cs}}$ (the center of the z-coordinate of the contact surface) in Eqs. (2.11) and (2.12), u_z^{cs} is derived as

$$\begin{aligned} u_z^{\text{cs}}(t) &\equiv u_{\text{th};z}^{\text{cs}} + u_{\text{fric};z}^{\text{cs}} \\ &\simeq \text{sgn}(z_{\text{cs}}-z_{\text{opt}})\frac{\alpha_{\text{th}}}{2A\rho c_p}W_{\text{abs}}^{\text{eff}}(t-t_0) + \frac{1}{2A\rho v_{\text{3D}}^{\text{L}}}\int_{-\infty}^{t} dt' F_{\text{fric};z}(t'), \end{aligned} \tag{2.13}$$

where $t_0 \equiv \frac{|z_{\text{cs}}-z_{\text{opt}}|}{v_{\text{3D}}^{\text{L}}}$ represents the time for the L-modes traveling from the center of the absorbed optical power to the center of contact surface.

To evaluate u_z^{cs}, the friction force needs to be specified. As is explained in the main text, the friction force can be determined by recognizing that

- *From a microscopic wave picture, the friction force behaves as a fence resisting the elastic waves from passing through the contact surface. In the static regime, the static friction force is large enough to totally reflect back the incident elastic waves and thus nullify mechanical oscillations on the contact surface (that is, the incident and reflected elastic waves cancel each other out perfectly), thereby preserving the contact surface in the still state. On the contrary, in the dynamic regime, the elastic waves are so strong that even the maximum allowable static friction force—the so-called sliding resistance force denoted by F_{silde}—cannot nullify them on the contact surface. The plate thus slides.*

To confirm this wave-cancellation picture, we re-examine Fig. 1C in the main text, and perform additional numerical simulations that partition the total excited elastic waves into the separate contributions from the absorbed optical power and the friction force. As is shown in Fig. S5A, with $F_{\text{slide}} = 2.7\,\mu$N, the peak value of the absorbed optical power exceeds P_{TH}, so that the amplitude of the thermally-excited elastic waves is larger that of the friction-force-induced elastic waves, and, accordingly, the elastic waves can pass through the contact surface. On the contrary, increasing F_{slide} to $40\,\mu$N, the peak value of the absorbed optical power becomes lower than P_{TH}. Consequently, the thermally-excited and friction-force

elastic waves cancel out each other, and result in the zero transmission of the elastic waves through the contact surface, as shown in Fig. S5B.

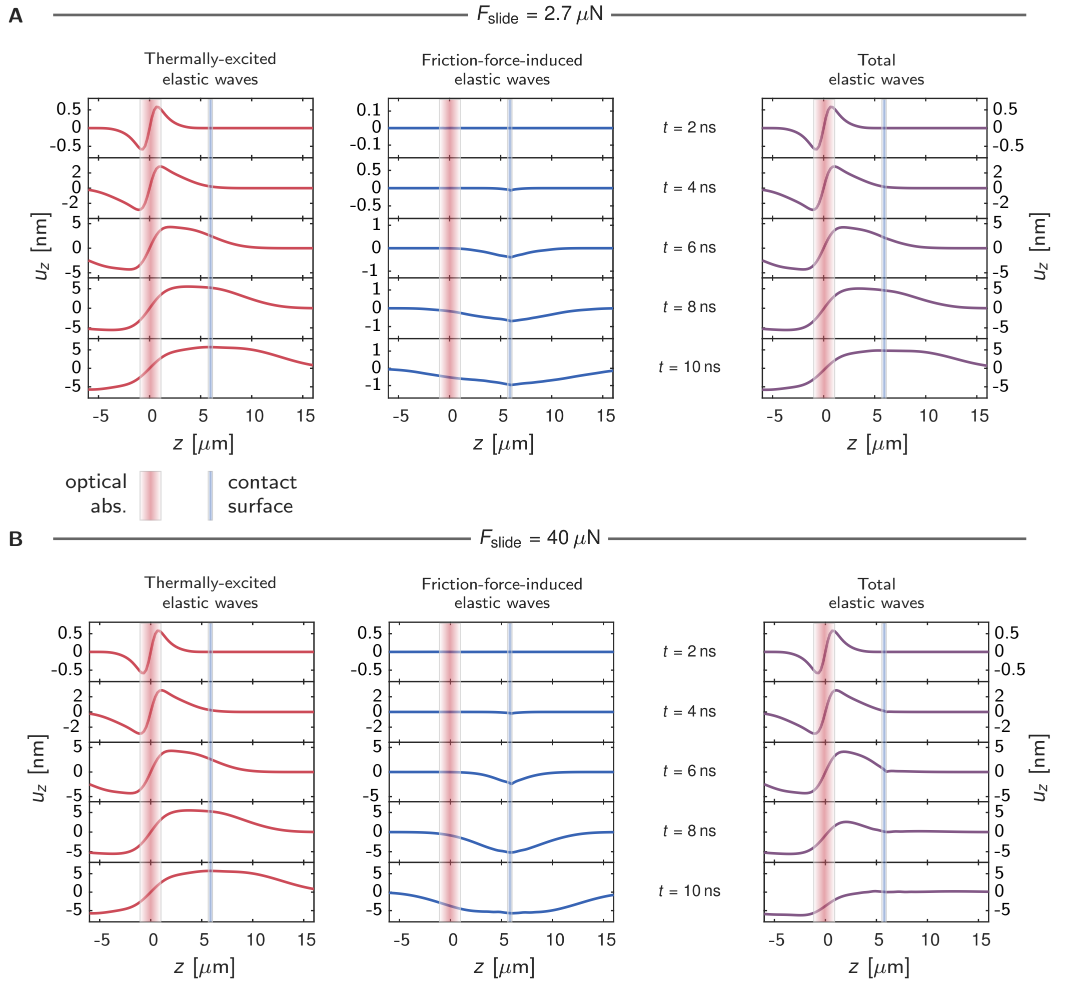

FIG. S5: **Numerical simulations of excited elastic waves, partitioning contributions from absorbed optical power and friction force**. The simulated system is as the same as studied in Fig. 1C in the main text with $F_{\text{slide}} = 2.7\,\mu\text{N}$ (**A**) and $F_{\text{slide}} = 40\,\mu\text{N}$ (**B**). The total elastic waves (rightmost panels) are the summation of thermally(optical absorption)-excited elastic waves (leftmost panels) and friction-force-induced ones (middle panels).

To concretize this physical picture, we first analyze the static regime wherein the contact surface is motionless. Accordingly, $du_z^{\text{cs}}/dt = 0$, which together with Eq. (2.13) gives the z-component of the friction force as follows

$$F_{\text{fric};z}(t) \simeq F_{\text{fric};z}^{\text{static}}(t) \quad \text{(static regime)}, \tag{2.14a}$$

with

$$F_{\text{fric};z}^{\text{static}} \equiv \text{sgn}(z_{\text{opt}} - z_{\text{cs}}) \frac{\alpha_{\text{th}} v_{3D}^{\text{L}} P_{\text{abs}}^{\text{eff}}(t - t_0)}{c_p}, \tag{2.14b}$$

where $P_{\text{abs}}^{\text{eff}} \equiv dW_{\text{abs}}^{\text{eff}}/dt = P_{\text{abs}} - P_{\text{leak}}$. Further, it is known that the magnitude of the static friction force is upper bounded by the sliding resistance F_{silde}, i.e., $|F_{\text{fric};z}^{\text{static}}| < F_{\text{silde}}$. This constrainment leads to that

$$|P_{\text{abs}}^{\text{eff}}(t - t_0)| < P_{\text{TH}} \tag{2.14c}$$

with

$$P_{\mathrm{TH}} = \frac{c_p F_{\mathrm{silde}}}{\alpha_{\mathrm{th}} v_{3\mathrm{D}}^{\mathrm{L}}}. \tag{2.15}$$

Apparently, the critical point, at which the plate starts moving, occurs when the constraint condition is just broken, i.e., $|P_{\mathrm{abs}}^{\mathrm{eff}}(t-t_0)| = P_{\mathrm{TH}}$. This observation renders P_{TH} a clear physical meaning: the minimum of the *instantaneous* power needed to overcome the sliding resistance. For this reason, P_{TH} is named as the *sliding threshold power*.

As the plate moves, it is subject to the dynamic friction force, whose magnitude generally has the same order as the sliding resistance. Taking this fact into account and for simplicity of analysis, we here assume that the magnitude of the dynamic friction force just equals the sliding resistance, i.e., approximating the dynamic friction force as

$$F_{\mathrm{fric};z}(t) \simeq \mathrm{sgn}\left[F_{\mathrm{fric};z}^{\mathrm{static}}(t)\right] F_{\mathrm{slide}} \quad \text{(dynamic regime)}, \tag{2.16a}$$

which is activated under the condition

$$|P_{\mathrm{abs}}^{\mathrm{eff}}(t-t_0)| > P_{\mathrm{TH}}. \tag{2.16b}$$

Moreover, exploiting the derived expressions of the friction force, the expression of the sliding displacement of the contact surface, Eq. (2.13), can be alternatively expressed as

$$u_z^{\mathrm{cs}}(t) \simeq \mathrm{sgn}(z_{\mathrm{cs}} - z_{\mathrm{opt}}) \frac{\alpha_{\mathrm{th}}}{2A\rho c_p} \int_{-\infty}^{t} dt' \mathrm{sgn}\left[P_{\mathrm{heat}}(t'-t_0)\right] \left(|P_{\mathrm{heat}}(t'-t_0)| - P_{\mathrm{TH}}\right) H(|P_{\mathrm{heat}}(t'-t_0)| - P_{\mathrm{TH}}) \tag{2.17}$$

with the term of the Heaviside step function [that is, $H(|P_{\mathrm{heat}}(t'-t_0)| - P_{\mathrm{TH}})$] explicitly specifying that the sliding occurs when $|P_{\mathrm{heat}}(t'-t_0)| > P_{\mathrm{TH}}$. From Eq. (2.17), we see that the sliding in principle is possible in both the heating period with $P_{\mathrm{heat}} > 0$ and the cooling period with $P_{\mathrm{heat}} < 0$. Two cases contribute to the sliding displacement in opposite directions as indicated by the term $\mathrm{sgn}\left[P_{\mathrm{heat}}(t'-t_0)\right]$. Referring to our problem, in the cooling period, the tiny contact surface between the plate and the substrate restricts the cooling efficiency significantly (see Fig. S10) and results in a negligible cooling power, so that the plate cannot slide. On the contrary, in the heating period, the noticeable absorbed optical power P_{abs} (with $P_{\mathrm{abs}}^{\mathrm{eff}} \simeq P_{\mathrm{abs}}$) can easily exceed P_{TH}, thereby driving the sliding of the plate. In view of this, we can safely replace P_{heat} with P_{abs} in Eq. (2.17) and obtain that

$$u_z^{\mathrm{cs}}(t) \simeq \mathrm{sgn}(z_{\mathrm{cs}} - z_{\mathrm{opt}}) \frac{\alpha_{\mathrm{th}}}{2A\rho c_p} \int_{-\infty}^{t} dt' (P_{\mathrm{abs}}(t'-t_0) - P_{\mathrm{TH}}) H(P_{\mathrm{abs}}(t'-t_0) - P_{\mathrm{TH}}). \tag{2.18}$$

Equations (2.13)—(2.18) summarize the main results of this section. Even though a few approximations are made to derive these equations, they show a good predictive accuracy as validated in Fig. 1C in the main text.

4. Attenuation effects

In the above derivations, the elastic attenuation is ignored. However, in reality, the attenuation always exists due to the elastic coupling between the gold nanoplate and the environment, in addition to the intrinsic damping mechanisms, such as lattice defects. To account for the attenuation effects, we here introduce a phenomenological model, which assumes that the elastic waves have frequency-independent life time, denoted by τ_{ac}. This assumption amounts to adding a constant imaginary part $1/(2\tau_{\mathrm{ac}} v_{3\mathrm{D}}^{\mathrm{L}})$ to $\beta_{3\mathrm{D}}^{\mathrm{L}}$. In this way, the attenuation effects can be effectively, conveniently incorporated. For instance, the sliding power threshold changes to

$$P_{\mathrm{TH}} = \frac{c_p F_{\mathrm{silde}}}{\alpha_{\mathrm{th}} v_{3\mathrm{D}}^{\mathrm{L}}} e^{t_0/(2\tau_{\mathrm{ac}})}, \tag{2.19}$$

which is multiplied by a factor of $e^{t_0/(2\tau_{\rm ac})}$ in comparison with the lossless case. Equation (2.19) is just Eq. (1) in the main text.

3. ROTATION DISPLACEMENT OF GOLD PLATES AROUND MICRO-FIBERS: THEORETICAL ANALYSIS

In this section, we theoretically analyze the rotation movement of gold plates around micro-fiber, as has been observed in our experiments. **The results here supplement the discussions attached to Fig. 4C in the main text.**

Figure S6A sketches the problem under investigation. The theoretical analysis here below is a direct generalization of the theory developed in Sec. 2.C by additionally taking multiple reflected elastic waves into account. The reflection spectrum of the fundamental L-modes is computed with COMSOL Multiphysics, as shown in Fig. S7. The results show that, at low frequencies, the reflection coefficients approximately equal to 1.

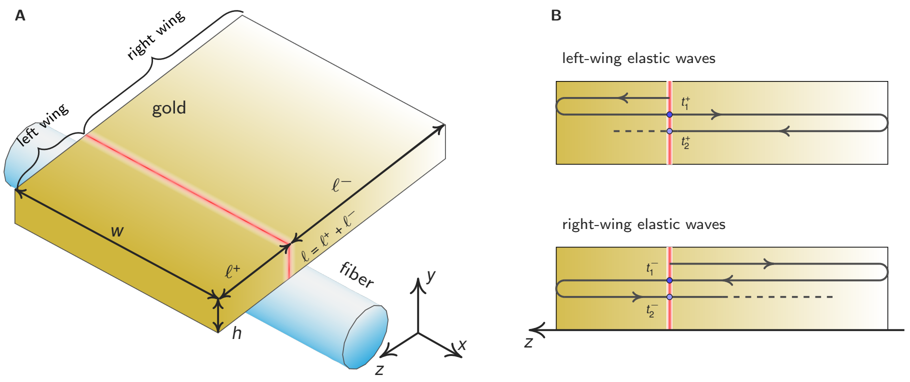

FIG. S6: **A**. Sketch of the experimental setup: a gold plate is placed on top of a micro-fiber. **B.** Excited elastic waves are classified into left- and right-wing waves, which initially propagate towards the left- and right-wings, respectively, before reflecting at two ends of the plate in the z−direction.

Effects of multiple reflections—The thermally-excited L−modes in the plate are classified into the left- and right-wing waves, according to their initial propagation directions towards the left- and right-wing sides, respectively (see Fig. S6B). The longitudinal components of the elastic displacement vectors carried by left- and right-wing waves are in the opposite directions. Specifically, their initial directions, before they reach the ends of the plate, are parallel to the wave propagation directions, i.e., pointing towards the left- and right-wing sides, respectively, as implied in Eq. (2.11). After reflections, the L-modes reverse their propagation directions, while the longitudinal components of the elastic displacement vectors do not change the sign because the reflection coefficients equal to 1 (under the long-wavelength approximation; see Fig. S7). Therefore, the left- and right-wing waves continuously drive the pate to rotate towards the left- and right-wing sides, respectively. Taking the aforementioned multiple reflections into account and employing Eqs. (2.11) and (2.12), the rotation displacement of the contact surface of the gold plate, denoted by $u^{\rm cs}_{\rm rot}$, is derived as follows

$$u^{\rm cs}_{\rm rot}(t) = \underbrace{u^{\rm cs}_{{\rm th};z}(t)}_{\text{thermal contribution}} + \underbrace{u^{\rm cs}_{{\rm fric};z}(t)}_{\text{friction-force contribution}} \tag{3.1a}$$

with

$$u^{\rm cs}_{{\rm th};z}(t) \simeq \frac{\alpha_{\rm th}}{2A\rho c_p}\sum_{n=1}^{\infty} \underbrace{W^{\rm eff}_{\rm abs}\left(t-t_n^+\right)e^{-t_n^+/(2\tau_{\rm ac})}}_{\text{left-wing waves}} - \underbrace{W^{\rm eff}_{\rm abs}\left(t-t_n^-\right)e^{-t_n^-/(2\tau_{\rm ac})}}_{\text{right-wing waves}}, \tag{3.1b}$$

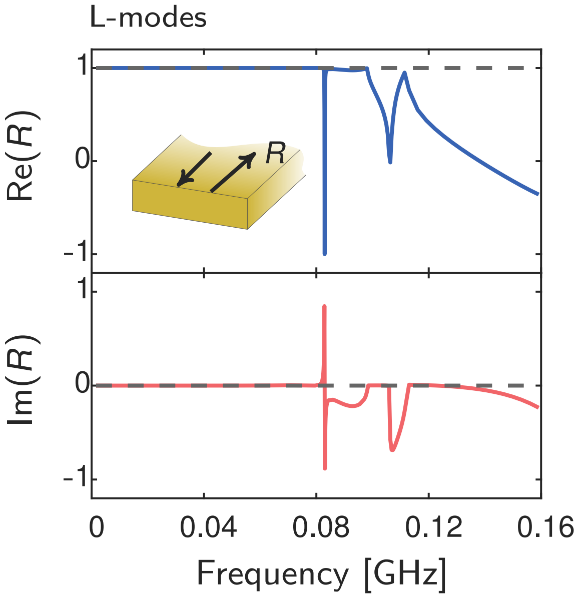

FIG. S7: **Reflection spectrum of fundamental *L*-modes**. The cross section of the studied gold plate has a width of 10 μm and a thickness of 30 nm. The numerical results (solid lines) are obtained with the COMSOL Multiphysics. The long-wavelength-approximation (LWA) results (dashed lines) represent the limiting values of the reflection coefficients as frequency approaches zero. The reflection coefficient is defined as the ration between the longitudinal component of the reflected *L*-modes and that of the incident ones.

and

$$u_{\text{fric};z}(t) \simeq \frac{P_z(t)}{2A\rho v_{3\text{D}}^{\text{L}}} + \frac{1}{2A\rho v_{3\text{D}}^{\text{L}}} \sum_{n=1}^{\infty} P_z(t - t_n^+)e^{-t_n^+/(2\tau_{\text{ac}})} + P_z(t - t_n^-)e^{-t_n^-/(2\tau_{\text{ac}})}. \tag{3.1c}$$

Here $P_z(t) \equiv \int_{-\infty}^{t} dt' F_{\text{fric};z}(t')$; $t_n^{\pm} = [2(n-1)\ell + 2\ell^{\pm}]/v_{3\text{D}}^{\text{L}}$ if n is an odd number and $t_n^{\pm} = 2(n-1)\ell/v_{3\text{D}}^{\text{L}}$ if n is an even number, where $\ell^{\pm}$ denote the lengths of the left and right wings, respectively, and $\ell = \ell^+ + \ell^-$ is the total length of the plate. Physically, $t_n^{\pm}$ represent the propagation time that the left- and right-wing waves take to return to their initial positions after n times reflections (see Fig. S6B). The attenuation effects are approximately incorporated with the damping terms $e^{-t_n^{\pm}/(2\tau_{\text{ac}})}$.

The friction force can be determined by using the same routine as in Sec. 2.C.3. First, the static friction force, resulting from $du_z^{\text{cs}}(t)/dt = 0$, is given by

$$F_{\text{fric}}(t) = F_{\text{fric}}^{\text{static}}(t) \quad \text{(static regime)}, \tag{3.2a}$$

with

$$F_{\text{fric}}^{\text{static}}(t) \equiv -\frac{\alpha_{\text{th}} E}{2A\rho^2 c_p \left(v_{3\text{D}}^{\text{L}}\right)^2} \sum_{n=1}^{\infty} P_{\text{abs}}^{\text{eff}}\left(t - t_n^+\right) e^{-t_{\text{tra;n}}^+/(2\tau_{\text{ac}})} - P_{\text{abs}}^{\text{eff}}\left(t - t_n^-\right) e^{-t_{\text{tra;n}}^-/(2\tau_{\text{ac}})}$$
$$- \frac{1}{2A\rho v_{3\text{D}}^{\text{L}}} \sum_{n=1}^{\infty} F_{\text{firc};z}(t - t_{\text{tra;n}}^+)e^{-t_n^+/(2\tau_{\text{ac}})}, \tag{3.2b}$$

and constrained by

$$|F_{\text{fric}}^{\text{static}}(t)| < F_{\text{slide}}.$$

In the dynamic regime, the friction force is given by

$$F_{\rm fric}(t) = {\rm sgn}\left[F^{\rm static}_{\rm fric}(t)\right] F_{\rm slide}. \quad \text{(dynamic regime)}, \tag{3.3}$$

with

$$|F^{\rm static}_{\rm fric}(t)| \geq F_{\rm slide}. \tag{3.4}$$

Strong-attenuation approximation—Equations (3.1)–(3.4) characterize the rotation movement of gold plates. However, they are complicated due to the presence of the infinite series. Therefore, examining them to reveal the physics is not straightforward. To bypass this complication, we simplify Eqs. (3.1)–(3.4) by retaining the leading-order terms in these equations. This amounts to keeping the $n = 1$ terms of the series in Eq. (3.1b) and dropping all the series in Eq. (3.1c). Such an approximation physically corresponds to *the strong elastic attenuation case* wherein all high-order reflected waves are sufficiently damped and thus become negligible. Under the strong-attenuation approximation and approximating that $P^{\rm eff}_{abs} \simeq P_{abs}$, $u^{\rm cs}_{\rm rot}$ could be arranged in a similar form as Eq. (2.18) and is expressed as

$$\begin{aligned} u^{\rm cs}_{\rm rot}(t) \simeq & \frac{\alpha_{\rm th}}{2A\rho c_p}\int_{-\infty}^{t} dt'(P_{\rm abs}(t'-t_1^+) - P^+_{\rm TH})H\left[P_{\rm abs}(t'-t_1^+) - P^+_{\rm TH}\right] \\ & - \frac{\alpha_{\rm th}}{2A\rho c_p}\int_{-\infty}^{t} dt'(P_{\rm abs}(t'-t_1^-) - P^-_{\rm TH})H\left[P_{\rm abs}(t'-t_1^-) - P^-_{\rm TH}\right], \end{aligned} \tag{3.5}$$

with

$$P^{\pm}_{\rm TH} = \frac{c_p F_{\rm silde} e^{t_1^{\pm}/(2\tau_{\rm ac})}}{\alpha_{\rm th} v^{\rm L}_{\rm 3D}} \tag{3.6}$$

representing the sliding power threshold for the left- and right-wing waves, respectively. Apparently, if $\ell^+ < \ell^-$, $P^+_{\rm TH} < P^-_{\rm TH}$, leading to $u^{\rm cs}_z > 0$; on the contrary, if $\ell^+ > \ell^-$, we have $u^{\rm cs}_z < 0$. Therefore, the rotation direction points from the long wing to the short wing.

4. OPTICAL ABSORPTION IN GOLD-PLATE & MICRO-FIBER COUPLED SYSTEM

In our experiments, we investigate gold-plate and micro-fiber coupled system, wherein a gold plate is adhered to a micro-fiber. Micro-fibers with diameter about a few μm can support a large number of optical modes in the wavelength range of the employed super-continuum laser (between 450 nm and 2400 nm). Fig. S8A shows the dispersion relations of optical modes in a 1.8 μm-diameter micro-fiber surrounded by air. Among these modes, we here discuss fundamental $\rm HE^{x,y}_{11}$ modes and high-order $\rm HE^{x,y}_{12}$ modes, which have the intensity maximum at the center of the fiber core (see Fig. S8B for modal profiles of $\rm HE^{x,y}_{11}$ and $\rm HE^{x,y}_{12}$ modes) and, thus, can be efficiently excited by external laser pulses whose spatial profiles are generally Gaussian.

Gold plates absorb evanescent electric fields of optical modes and then generate heat. Figure S8C depicts the computed absorption spectra for a square gold plate—with side length 10 μm and thickness 30 nm—, which is symmetrically placed on a micro-fiber with diameter 1.8μm for the $\rm HE^{x,y}_{11}$ and $\rm HE^{x,y}_{12}$ modes as incident waves. The results show that the $\rm HE^{y}_{11}$ and $\rm HE^{y}_{12}$ modes are more efficiently absorbed than the $\rm HE^{x}_{11}$ and $\rm HE^{x}_{12}$ modes. This is due to that the dominant component of electric fields of the $\rm HE^{y}_{11}$ and $\rm HE^{y}_{12}$ modes is perpendicular to the surface of the plate, which enables efficient excitations of plasmonic modes in the plate, thereby benefiting optical absorption through field-enhancement effects. While for the $\rm HE^{x}_{11}$ and $\rm HE^{x}_{12}$ modes, the dominant component of electric fields is instead parallel to the surface of the plate, unfavorable for plasmonic excitations. Moreover, the plasmonic excitations due to the $\rm HE^{y}_{11}$ and $\rm HE^{y}_{12}$ incidences result in subwavelength localizations of electric fields in the plate along the x and y directions, as shown in Fig. S8D.

We measured the absorption spectra of a gold-plate and micro-fiber coupled system, as shown in Fig. S9C. Comparing with the simulation results, the measured absorptance is higher. This is attributed to that the size of the measure gold plate is larger than the simulated one. Moreover, it should be noted that the measured spectra are contributed from multi-modes of the micro-fiber and thus cannot be directly,

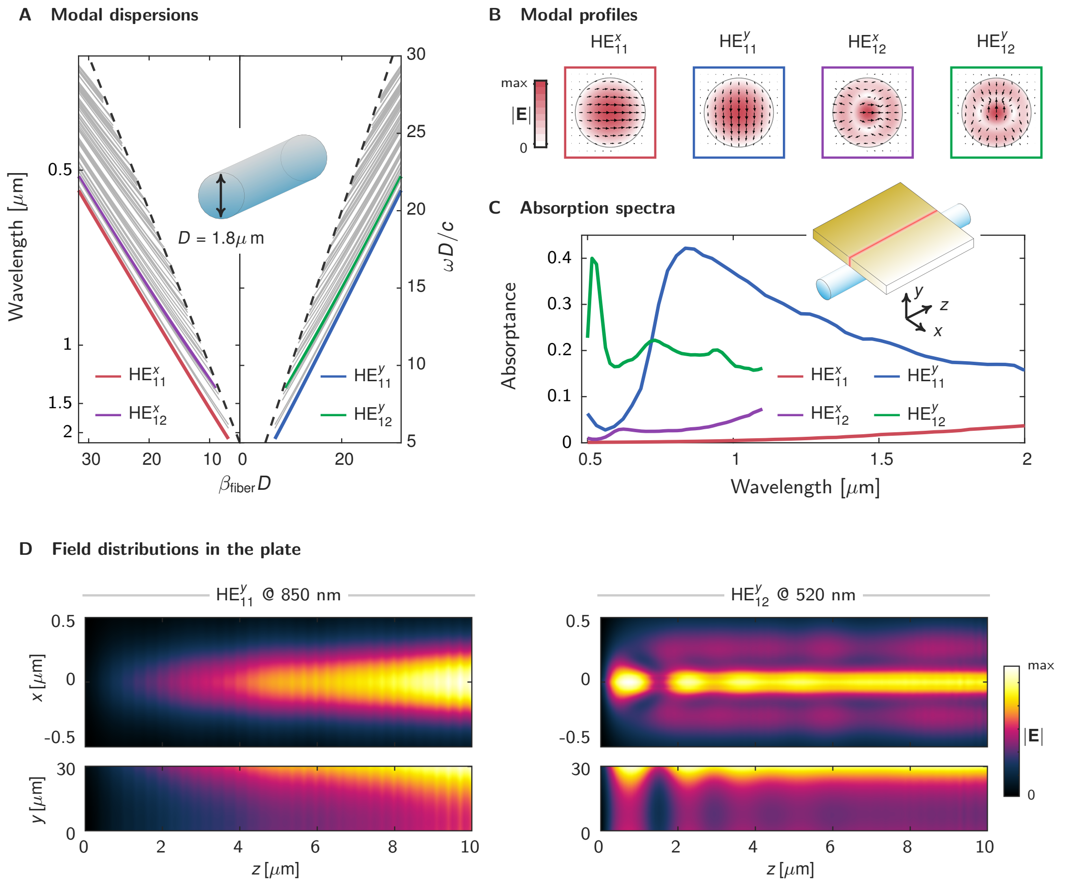

FIG. S8: **Optical absorption in gold-plate & micro-fiber coupled system. A.** Dispersion relations of optical waveguide modes of a 1.8 μm-diameter micro-fiber in air. The refractive index of the micro-fiber is 1.46. **B.** Modal profiles of fundamental $\mathrm{HE}_{11}^{x,y}$ modes and high-order $\mathrm{HE}_{12}^{x,y}$ modes. **C.** Absorption spectra of $\mathrm{HE}_{11}^{x,y}$ and $\mathrm{HE}_{12}^{x,y}$ modes. The gold plate has a square base shape with side length 10 μm and thickness 30 nm. **D.** Distributions of electric field strength in the $x-z$ and $y-z$ planes through the center of the plate for the incidences of the HE_{11}^{y} (left) and HE_{12}^{y} (right) modes. The wavelengths are at 850 nm and 520 nm for two modes, respectively, which correspond to the spectral positions of their absorption peaks (**B**).

quantitatively mapped to the simulated results of single individual modes in Fig. S9C.

Remarks—Even though we can precisely compute electromagnetic interactions between gold plates and individual optical modes of micro-fibers, it remains challenging to estimate the joint contributions from all modes, because experimentally quantifying coupling efficiencies of external laser pulses into various optical modes in the wide wavelength range of the used super-continuum laser is difficult. In view of this, in the below heat-elastic simulations that demand spatial distributions of absorbed optical power as an indispensable input, we retreat to a simple expression, Eq. (5.1), as an approximate representation.

5. HEATING AND COOLING DYNAMICS IN GOLD-PLATE & MICRO-FIBER COUPLED SYSTEM

In this section, we numerically examine the thermal heating and cooling dynamics in gold-plate & micro-fiber coupled system. Figure S10A sketches the simulation domain comprising of a gold plate, a micro-fiber and air background (their geometrical dimensions are summarized in the caption of the figure). Both the axial length of the micro-fiber and the background size are set large enough such that further increasing their values does not change simulation results significantly. The absorbed optical power

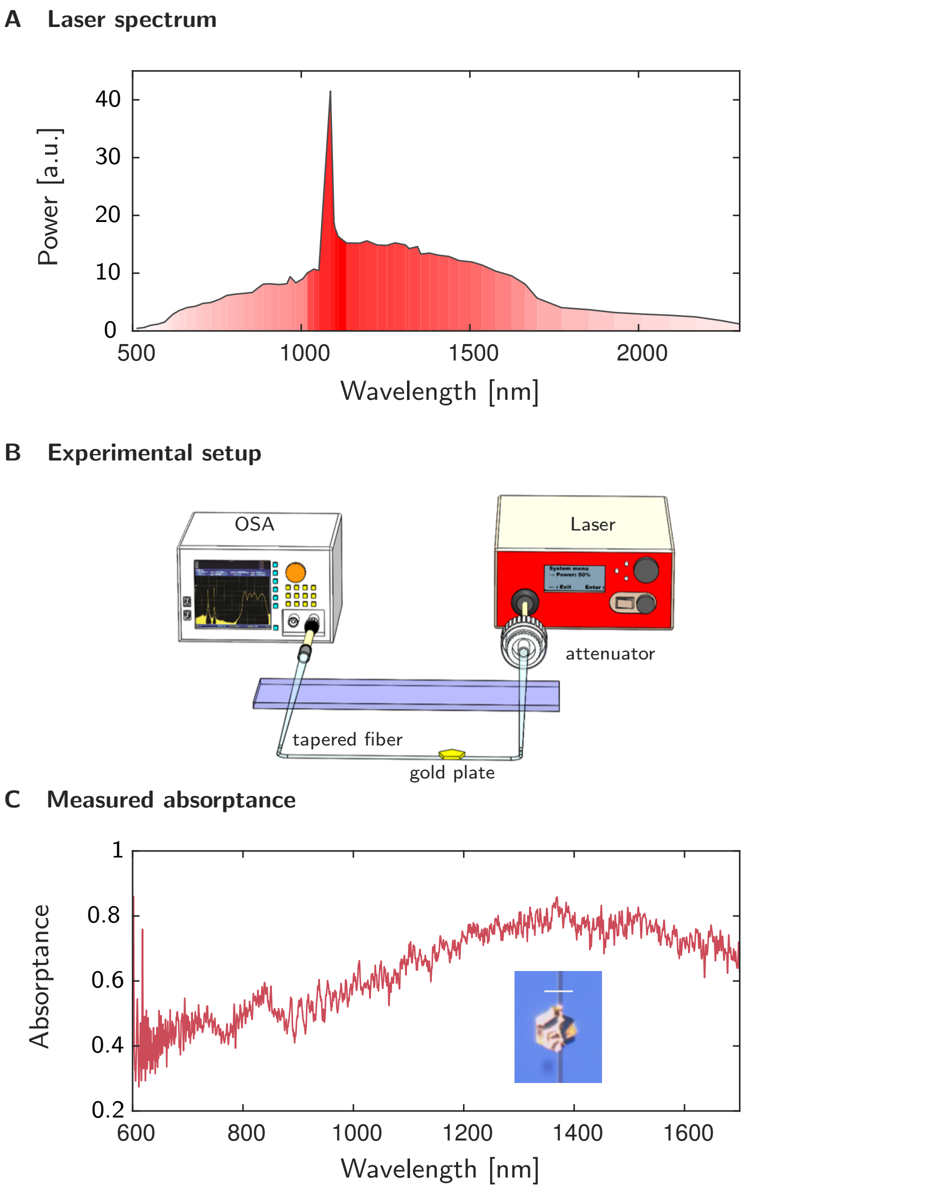

FIG. S9: **A.** Power spectrum of the employed supercontinuum laser. **B.** Sketch of experimental setup for measuring absorption spectra. **C.** Measured absorption spectra of a hexagonal gold-plate & micro-fiber coupled system. Inset: an optical image of the gold plate on the micro-fiber (scale bar, 15 μm).

density Q_{abs} is set as

$$Q_{\text{abs}}(\mathbf{r};t) = \overbrace{P_{\text{peak}}}^{\text{peak power}} \underbrace{\frac{1}{\sqrt{\pi}\tau_p} e^{-(t-t_0)^2/\tau_p^2}}_{\text{temporal distribution}} \underbrace{\frac{1}{wh} \frac{1}{\sqrt{\pi}\ell_p} e^{-(z-z_{\text{cs}})^2/\ell_p^2}}_{\text{spatial distribution}}, \tag{5.1}$$

where $t_0 = 3\,\text{ns}$, $\tau_p = 1.5\,\text{ns}$, $\ell_p = 250\,\text{nm}$, $z_{\text{cs}} = 0$ and $P_{\text{peak}} = 100\,\text{mW}$; w and h denote the width and the thickness of the plate, respectively (see the inset in Fig. S10A).

Figure S10B shows the temperature at the center of the plate (upper panel), the net heat energy $W^{\text{eff}}_{\text{abs}}$ stored in the plate (middle panel) and the effective heat power $P^{\text{eff}}_{\text{abs}} \equiv dW^{\text{eff}}_{\text{abs}}(t)/dt$ (lower panel), as functions of time. The results show that the plate is rapidly heated within the short period of the optical absorption pulse, and then slowly cooled by transferring heat into the air background and the micro-fiber.

The cooling efficiency is low due to (i) low thermal conductivity of air and (ii) tiny contact surface between the plate and the micro-fiber. As a result, P_{abs}^{eff} in the cooling period has a rather small magnitude and its maximum is only about 5 mW, about 5% of the absorption peak power. The complete cooling takes more than 10 μs.

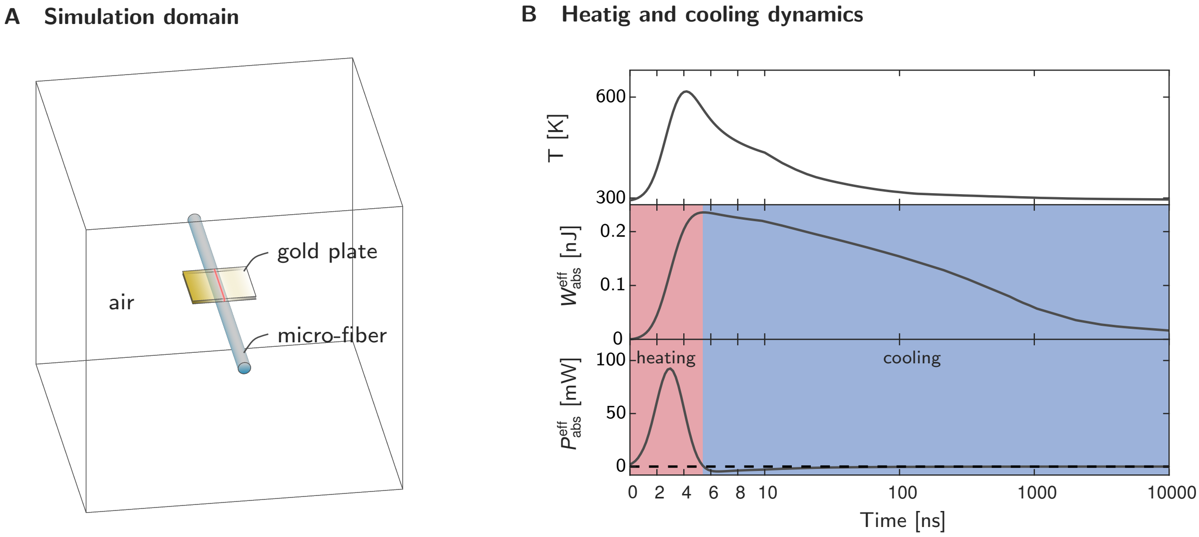

FIG. S10: **Thermal dynamics in gold-plate & micro-fiber coupled system driven by a single optical pulse. A**. Sketch of the simulation domain. A square gold plate with side length 10 μm and thickness 30 nm is placed on top of a micro-fiber with diameter 1.8 μm and length 50 μm. The two wings of the plate separated by the micro-fiber are of equal length. An air cubic box with side length 50 μm surrounds the plate and the micro-fiber. The driven optical pulse is specified in Eq. (5.1). **B.** Temperature at the center of the plate (upper panel), net heat energy W_{heat} (middle panel) and heat power P_{heat} (lower panel) of the plate, as functions of time.

6. COUPLED HEAT-ELASTIC SIMULATIONS

In this section, we present more results on modelling locomotion of gold plates around micro-fibers driven by optical pulses, **supplementing Fig. 4 and the associated discussions in the main text**.

The numerical modelling amounts to solving the coupled heat-conduction and elastic equations:

$$\rho c_p \frac{\partial \delta T(\mathbf{r};t)}{\partial t} - \boldsymbol{\nabla} \cdot K \boldsymbol{\nabla} \delta T(\mathbf{r};t) = Q_{abs}(\mathbf{r};t), \tag{6.1a}$$

$$\boldsymbol{\nabla} \times \boldsymbol{\nabla} \times \mathbf{u}(\mathbf{r};t) - \frac{2(1-\mu)}{1-2\mu} \boldsymbol{\nabla}\boldsymbol{\nabla} \cdot \mathbf{u}(\mathbf{r};t) + \frac{2\rho(1+\mu)}{E} \frac{\partial^2 \mathbf{u}(\mathbf{r};t)}{\partial t^2} = -\alpha_{th} \frac{2(1+\mu)}{(1-2\mu)} \boldsymbol{\nabla} \delta T(\mathbf{r};t) + \frac{2(1+\mu)}{E} \mathbf{f}_{fric}(\mathbf{r};t). \tag{6.1b}$$

The thermal-mechanical parameters used here are summarized in Table I. The expression of absorbed optical power Q_{abs} is given in Eq. (5.1). The simulations are performed with COMSOL Multiphysics.

In experiments, we observe that gold plates move spirally around micro-fibers. The spiral motion has two fundamental degrees of freedom: rotation and translation in the azimuthal and axial directions of micro-fibers, respectively, which will be discuss separately below.

A. Rotation

We below present the supplementary results for Fig. 4C in the main text. Figure S11A contrasts the rotation displacements of the contact surface and the centroid of the studied gold plate in the rotation

	specific heat capacity, c_p [J/(kg·K)]	thermal conductivity, K [W/(m·K)]	coefficient of linear thermal expansion,α_{th} [1/K]
gold	120	110	31.5×10^{-6}
silica	730	1.4	0.5×10^{-6}
air	1000	0.03	

	Young's modulus, E [Pa]	Possion's ratio, μ	density, ρ [kg/m^3]
gold	70×10^9	0.44	19300
silica	70×10^9	0.17	2200

TABLE I: **Thermal-mechanical parameters used in the numerical simulations.**

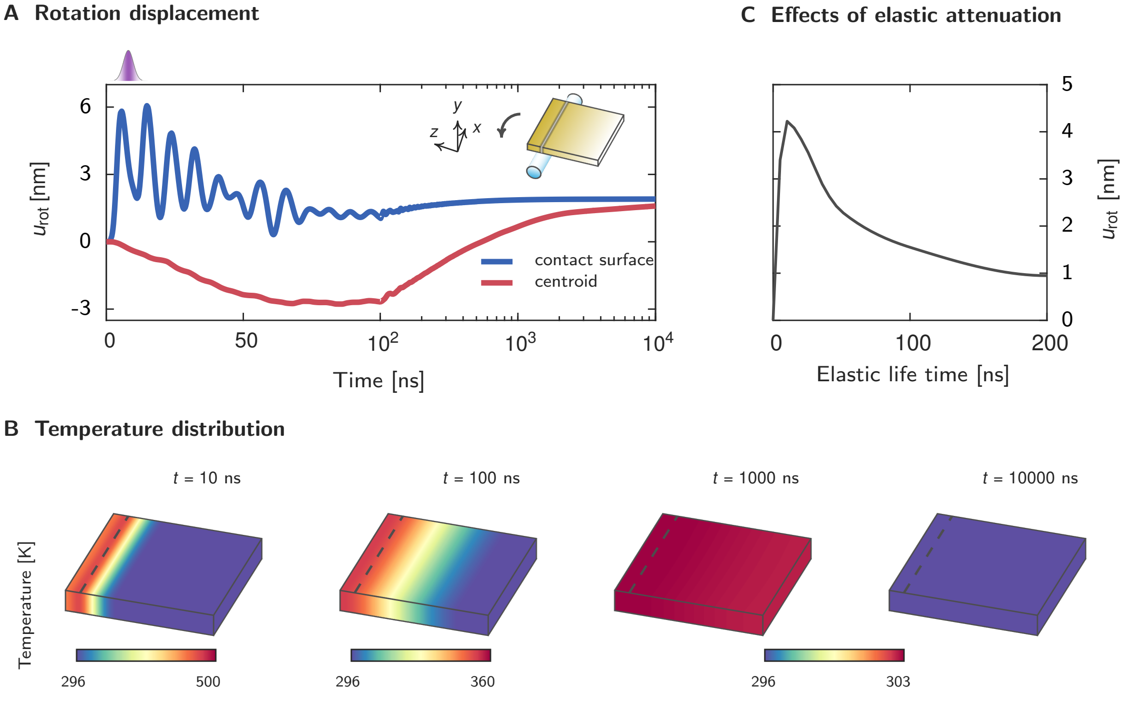

FIG. S11: **Rotation dynamics in gold-plate & micro-fiber system, supplemental to Fig. 4C in the main text**. **A.** Temporal evolution of rotation displacements of the contact surface and the centroid of a gold plate driven by a single optical pulse. **B.** Temperature distribution in the plate at different time. **C.** Stabilized rotation displacement of the plate driven by an optical pulse as a function of the life time of elastic waves. The gold square plate with side length $10\,\mu$m and thickness 30 nm is placed on a micro-fiber with diameter 1.8μm. The two wings of the plate are of unequal length of $1\,\mu$m and $9\,\mu$m, respectively. The pulsed optical absorption is specified in Eq. (5.1). The line-shaped contact surface between the plate and the micro-fiber is smooth along the axial direction of the micro-fiber and it supports a frictional sliding resistance of $1.5\,\mu$N. The life time of elastic waves is $\tau_{\mathrm{el}} = 20\,$ns in **A**-**B** and is varied in **B**.

direction as functions of time. Interestingly, it is observed that, at earlier times (smaller than one hundred nanoseconds) when the plate slides noticeably, the centroidal displacement (i.e., the average displacement of the plate) and the contact-surface displacement show opposite signs. This can be understood by analyzing the friction force (see the middle panel in Fig. 4C in the main text). In specifics, the friction force points in the opposite direction with the contact-surface displacement, while the centroid of the

plate moves in the direction of the friction force due to Newton's second law. Then, as time increases such that the elastic waves in the plate are significantly damped, the contact surface ceases sliding. At the mean time, the centoridal displacement gradually reverses its sign and finally approaches the value of the contact-surface displacement. This is due to the existence of the static friction force that points in the same direction as the accumulated contact-surface displacement, thereby preventing the contact surface from returning to its initial position and additionally dragging the centroidal displacement towards the direction of the contact-surface displacement. Figure S11B plots the temperature distribution in the plate at different time.

Figure S11C depicts the stabilized rotation displacement driven by a single optical pulse as a function of life time of elastic waves. It is observed that, as the elastic life time increases (i.e., the attenuation decreases), the magnitude of the displacement initially increases benefiting from the reduced attenuation, and then decreases as a result of the restored balance between the counter-propagating waves in the plate (see Sec. 3).

B. Translation

1. Supplementary results for Fig. 3D

We below present the supplementary results for Fig. 3D in the main text. Figure S12A contrasts the translation displacements of the contact surface and the centroid of a gold plate in the axial direction of a micro-fiber as functions of time. The centrodial displacement here shows similar features as in the rotation case in Fig. S11, which can be understood by analyzing the friction force (middle panel in Fig. 4D in the main text; following the same reasonings as the rotation case). Figures S12B plots the temperature distribution in the plate at different time. Figures S12C shows the stabilized translation displacement driven by a single pulse as a function of life time of elastic waves, consistent with the results in the rotation case.

2. Effects of asymmetrical distribution of absorbed optical power on translation

In Fig. 3D in the main text and Fig. S12, we set that optical absorption power distributes uniformly along the axial direction of micro-fibers. This setting is derived from the experimental observation that the translation direction is independent of light propagation direction, and the contact asymmetry is identified as the key factor determining the translation direction of the plate (see the associated discussions in the main text).

Detaching our considerations from the experimental facts, one may wonder, if the surface curvature of gold plates is negligible such that the contact surface is "flat" along the axial direction of micro-fibers, how do plates translate? As is discussed in the main text, to enable the translation of plates, the reflection symmetry of the system in the axial direction of micro-fibers needs to be broken. Apparently, in the absence of the contact asymmetry, this requirement can also be achieved by the asymmetrical distribution of the absorbed optical power, which is termed as optical asymmetry in the following discussions.

To model the optical asymmetry, we modify Eq. (5.1) to

$$Q_{\rm abs}(\mathbf{r};t) = P_{\rm peak}\frac{1}{\sqrt{\pi}\tau_p}e^{-(t-t_0)^2/\tau_p^2}\frac{1}{wh}\frac{1}{\sqrt{\pi}\ell_p}e^{-(z-z_{\rm cs})^2/\ell_p^2}\underbrace{f_{\rm asy}e^{-(x-x_{\rm asy})^2/w_p^2}}_{\text{asymmetrical distribution}}. \tag{6.2}$$

Here, the optical asymmetry is introduced by a nonzero value of $x_{\rm asy}$ (the center coordinate of the contact surface in the axial direction of micro-fibers is $x = 0$); $f_{\rm asy}$ is the normalization factor such that $\int_{-w/2}^{w/2} dx f_{\rm asy} e^{-(x-x_{\rm asy})^2/w_p^2} = 1$.

In the following numerical study, we change the simulation settings of Fig. S12 by (i) employing Eq. (6.2) to represent the pulsed optical absorption and setting $x_{\rm asy} = 5\,\mu$m and $w_p = 5\,\mu$m, and (ii) removing the contact asymmetry. To examine the translation dynamics, we select two points locating on the front and back edges of the plate in the x-direction (see Fig. S13A). Note that the magnitude of absorbed optical power decreases monotonically from the front edge of the plate to the back edge (see the upper panel in Fig. S13B).

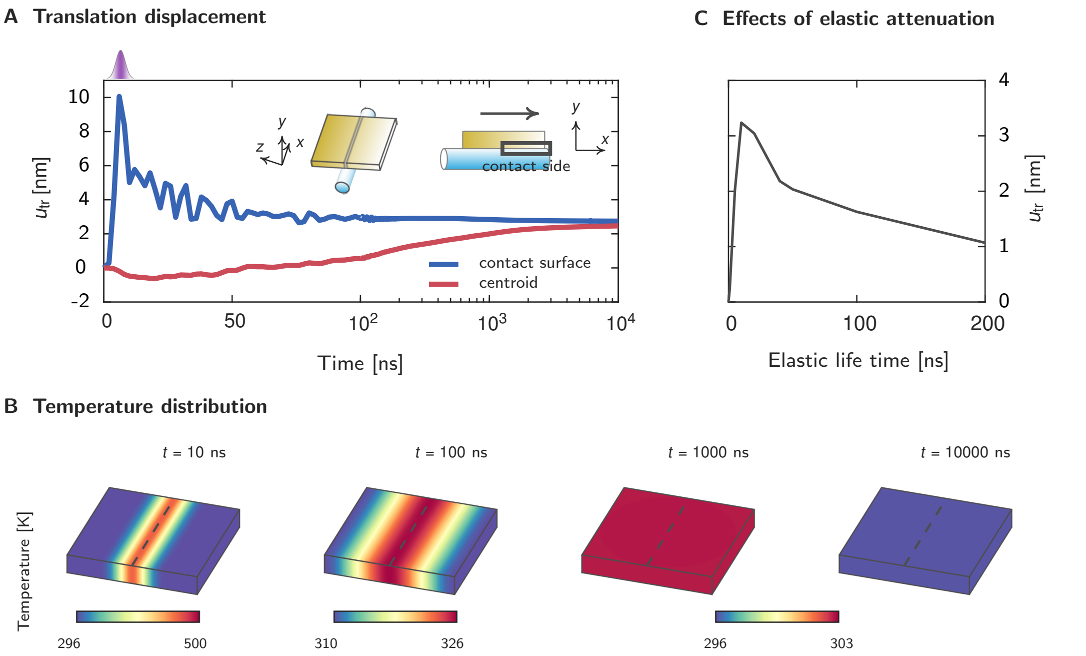

FIG. S12: **Translation dynamics in gold-plate & micro-fiber system, supplemental to Fig. 4D in the main text**. **A.** Temporal evolution of translation displacements of the contact surface and the centroid of a gold plate driven by a single optical pulse.**B.** Temperature distribution in the plate at different time. **C.** Stabilized translation displacement of the plate driven by a single optical pulse as a function of life time of elastic waves. The gold square plate with side length 10 μm and thickness 30 nm is placed on a micro-fiber with diameter 1.8 μm. The two wings of the plate are of equal length. The pulsed optical absorption is specified in Eq. (5.1). The line-shaped contact surface between the plate and the micro-fiber occupies the half part of the ideal smooth touching line, mimicking the contact asymmetry induced by the surface curvature of the plate; the frictional sliding resistance is 1.5 μN. The life time of elastic waves is $\tau_{\rm el}$ = 20 ns in **A**-**B** and is varied in **B**.

As shown in Fig. S13C, the selected front and back points translate in the opposite directions initially, and the magnitude of the translation displacement of the front point is noticeably larger than the back point. The latter observation is due to the temperature at the front edge is higher than the back one (see the lower panel in Fig. S13B), resulting in larger thermal expansion. As time increases, the difference of the translation displacements of two points decreases as a result of thermal contraction, and, finally, converge to the same value. Notably, in this process, the translation direction of the back point reverses from the negative x-direction to the positive x-direction, while that of the front point retains the positive x-direction unchanged. Therefore, the net translation direction points from the back point to the front point, (i.e., towards the side localizing more optical absorption power). Moreover, the lower panel of Figs. S13C shows the translation profiles of the plate at different times.

Figure S13D plots the stabilized translation displacement of the plate as a function of the parameter of the optical asymmetry, $x_{\rm asy}$. It is seen that, as $x_{\rm asy}$ varies from $-5\,\mu$m to $5\,\mu$m, the direction of the translation displacement reverses its sign.

In summary, the above results evidence that, in the absence of the contact asymmetry, the optical asymmetry can also enable the translation. In the presence of both the contact and optical asymmetries, the translation direction shall be determined by the dominant one. The investigations in this direction shall be discussed in a future paper by us.

7. FABRICATED GOLD PLATES

Figure S14 plots confocal laser scanning microscopy images of fabricated gold plates of different shapes.

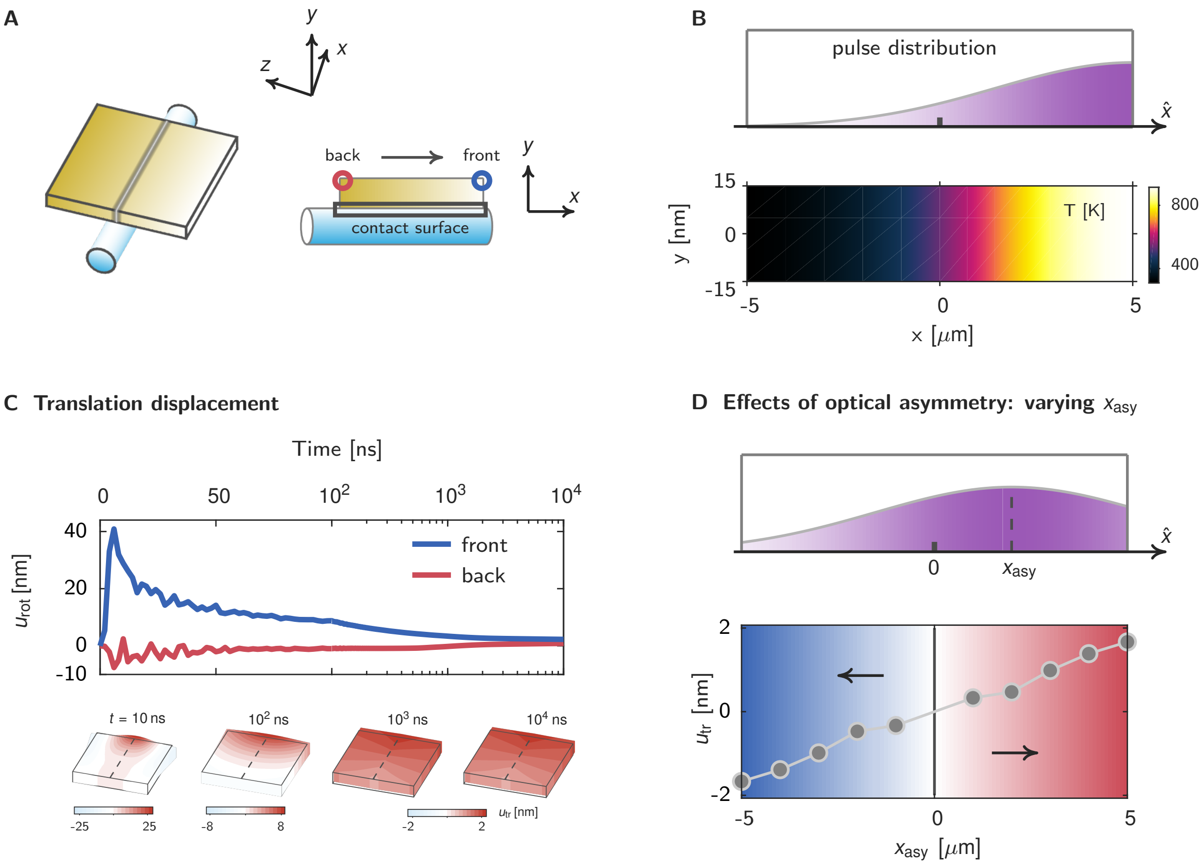

FIG. S13: **Effects of asymmetrical spatial distribution of optical absorption power on translation movement of gold plates.** **A.** Sketch of the problem. A gold square plate with side length 10 μm and thickness 30 nm is placed symmetrically with two equal-length wings on top of a micro-fiber with a diameter of 1.8 μm. The plate and the micro-fiber contacts each other smoothly along the axial direction of the micro-fiber and the frictional sliding resistance is 1.5 μN. The plate is driven by a pulsed optical absorption. The pulse is centered at 3 ns with 3-ns FWHM and its spatial asymmetry along the axial direction of the micro-fiber is parameterized by x_{asy}, see Eq. (6.2). **B.** Distribution profile of optical absorption power with $x_{\text{asy}} = 5\,\mu$m through the center of the gold plate in the axial direction of the micro-fiber (upper panel), and simulated temperature distribution at $t = 6$ ns (lower panel). Note that the temperature at the front edge of the plate is higher than the back edge because the optical absorption power localizes at the front edge. **C.** Temporal evolutions of translation displacements of two points on the front- and back-edges of the plate, respectively (upper panel), and translation profiles at different times (lower panel). The plate is driven by the pulsed optical absorption as shown in **B**. **D.** Stabilized translation displacement as a function of x_{asy}.

8. EXPERIMENTAL CHARACTERIZATIONS OF SPIRAL MOTION OF GOLD PLATES AROUND MICRO-FIBERS

A. Optical Images of Spiral Motion

Figure S15 demonstrates the recorded temporal sequential optical images of gold plates moving around micro-fibers spirally. The demonstrated gold plates here have different base shapes, including circle, rectangle, triangle and square (from up to down).

Technical remarks on determining rotation direction—The identification of the rotation direction needs to know relative positions between gold plates and micro-fibers at different times. This task however

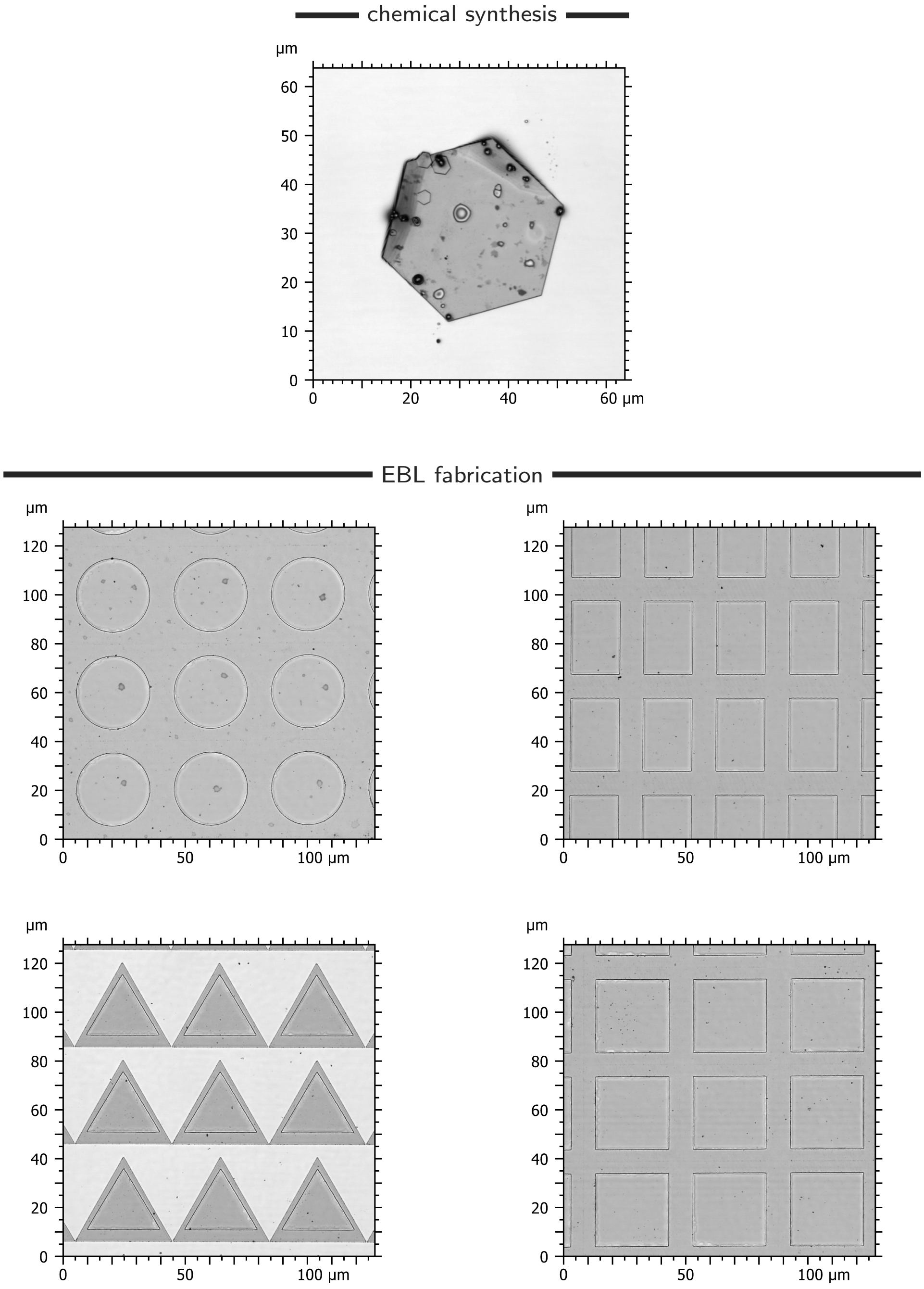

FIG. S14: **Confocal laser scanning microscopy images of fabricated gold plates**.

is not straightforward, because, in optical images, optical fibers with low refractive index are almost invisible when they overlap with non-transparent gold plates (see Fig. S15). Nevertheless, some fine features from the optical images can be exploited to specify the relative positions. Taking the triangle plate in Fig. S15 as an example, the optical image at $t = 0.72$ s, as zoomed in Fig. S16 A, show a clear line-shaped scattering pattern along the axial direction of the micro-fiber. This regular pattern is from light scattered by the gold plate. Further, we deduce that the gold plate locates below the micro-fiber. If not this scattering will be blocked by the plate and cannot be seen. Then, examining the optical image at $t = 0.96$ s, wherein the area of the plate is at the maximum and which is temporally close to the image at $t = 0.72$ s, we can conclude that the plate is just perpendicularly below the micro-fiber. Next, we examine

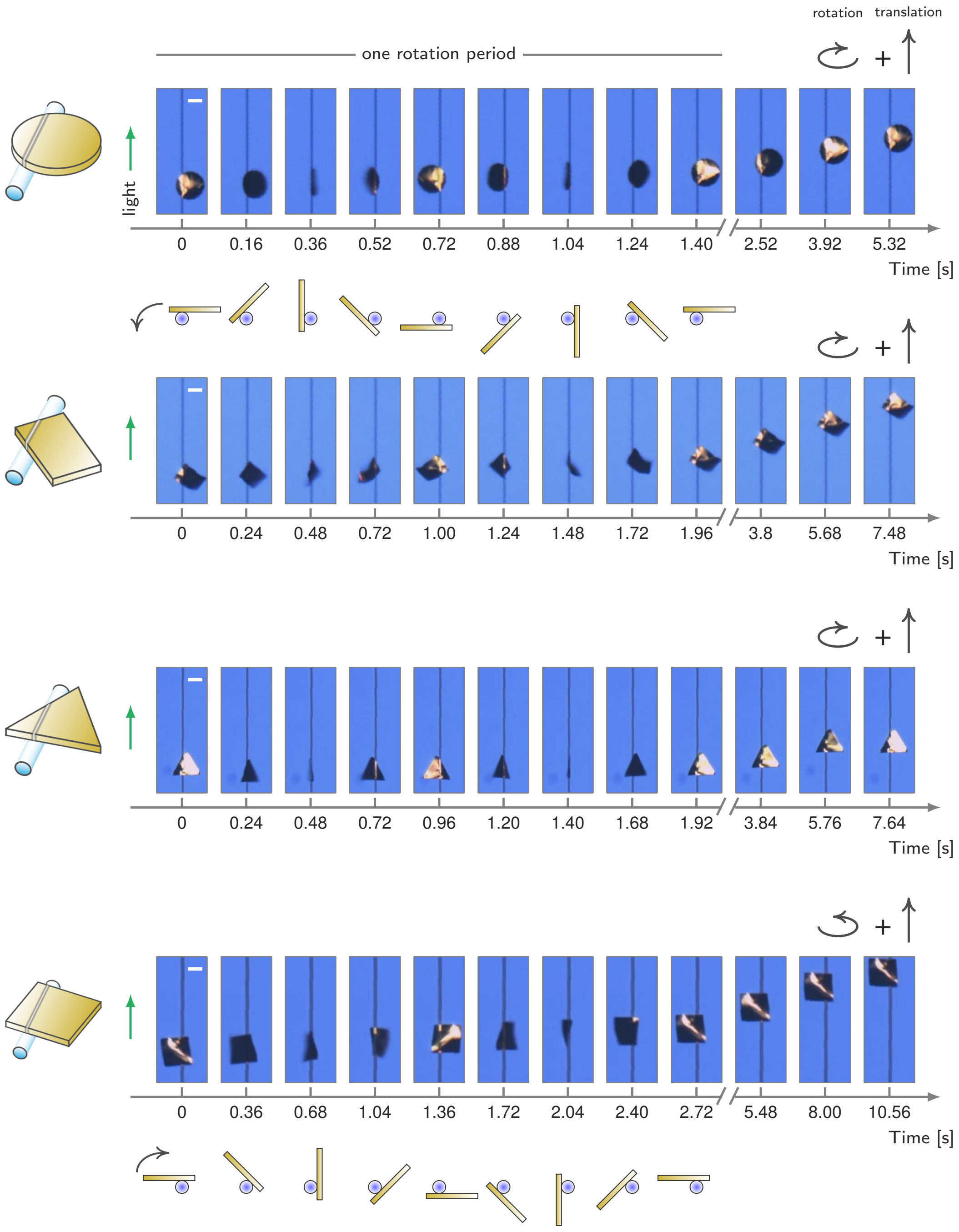

FIG. S15: **Sequential optical images of gold plates of various shapes spirally moving around micro-fibers.** All scalar bars represent 15 μm. The parameters of the laser source are as the same as in Fig. 2 in the main text.

the optical image at t = 1.48 s, as shown in Fig. S16B. The image clearly demonstrates the micro-fiber is on the left side of the plate. Consequently, for the optical image at t = 1.40 s (temporally close to optical image at t = 1.48 s), wherein the area of the plate is at the minimum, we deduce that the plate just touches the rightmost of the micro-fiber (see Fig. S16B). Knowing two specified positions, the rotation direction is determined unambiguously.

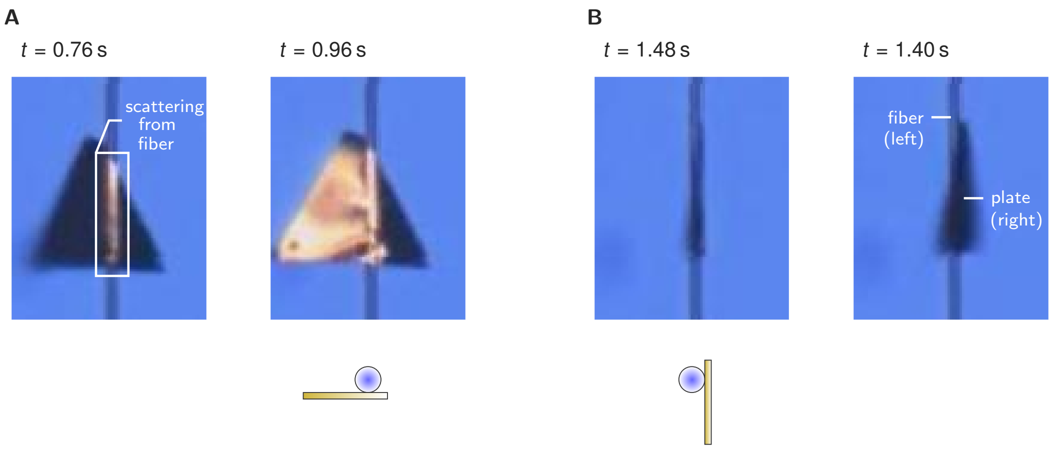

FIG. S16: **Identifying relative positions between a gold plate and a micro-fiber by examining optical images.**

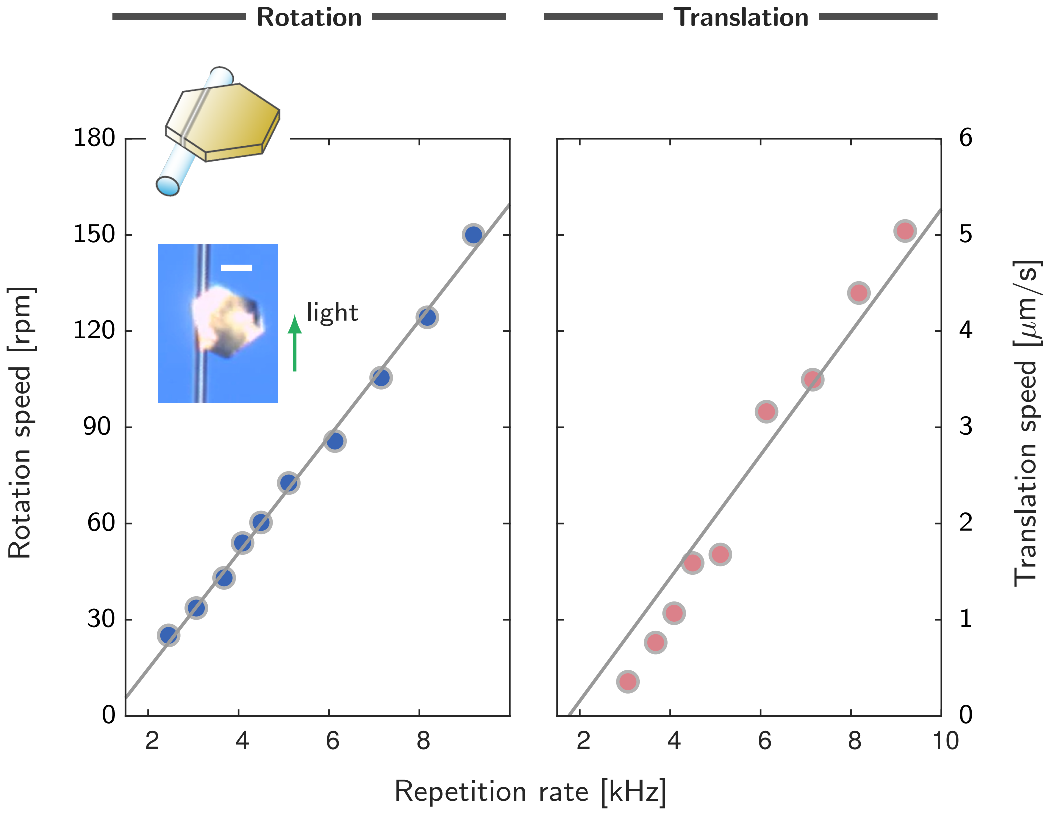

FIG. S17: **Rotation and translation speeds of a hexagonal gold plate spirally** moving around a micro-fiber as a function of repetition rate of laser pulses. Inset: optical image of the gold plate (scalar bar, 15 μm). The used super-continuum laser pulses here have 6.8–mW average power.

B. Controlling Motion Speed by Varying Repetition Rate of Laser Pulses

We measured the dependence of motion speeds of gold plates on the repetition rate of the used laser pulses. As shown in Fig. S17, both the rotation and translation speeds of a hexagonal plate increase (approximately) linearly as the repetition rate increases, evidencing that the spiral motion of the plate is driven by single individual pulse in a stepwise manner.

C. Manipulating Spiral Motion by Adjusting Relative Positions between Gold Plates and Micro-fibers

As has been discussed in the main text, the translation and rotation directions of gold plates depend on the asymmetries of their two wings and contact surfaces, respectively. Apparently, the wings' asymmetry can be conveniently controlled by adjusting the relative positions between plates and micro-fibers. However, for the contact asymmetry induced by the surface curvature of gold plates, the control is not straightforward, because the curvature features of plates are almost invisible under optical microscope, with which we adjust the positions of plates. Nevertheless, it is still possible to accidentally alter the contact asymmetry by randomly moving plates on micro-fibers. Figure S18 shows that by adjusting the positions of a gold plate on a micro-fiber, its motion directions as well as motion speeds can be adjusted . Notably, the rotation speeds decrease significantly as two wings become more asymmetric, consistent with Fig. 4E in the main text.

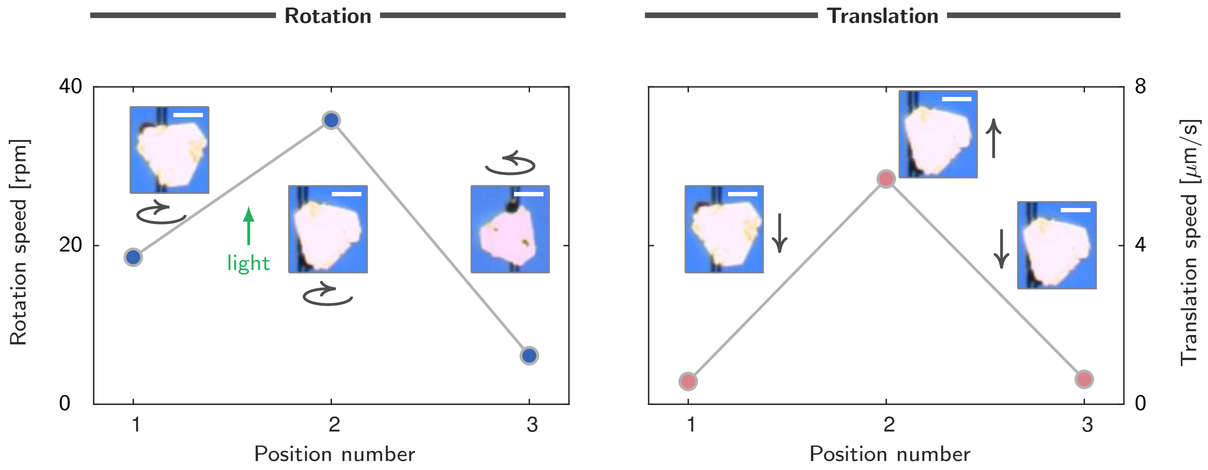

FIG. S18: **Varying spiral rotation of a gold plate by adjusting its relative position with respect to the contacted micro-fiber.** The parameters of the laser source are as the same as in Fig. 2 in the main text. All scale bars represent 15 μm.

D. Spiral Motion in a Vacuum Chamber

The experiments demonstrated in the main text and above in the Supplementary Information are performed in air. As is known, air molecules can exert two types of forces on their surrounded object: photophoretic force and drag force (i.e., air resistance). The photophoretic force is proportional to the air pressure and the temperature gradient on the object, with the magnitude estimated to be in the order of pN [2]. Differently, the drag force relates with the air density, the area and the velocity of the object [3], i.e., $F_{\mathrm{drag}} = C_D \rho A v^2/2$ with drag coefficient $C_D \sim 1$, air density ρ, object area A, and velocity v. Its magnitude in our case is in the order of 100 pN, considering the instantaneous velocity of the plate is ~1 m/s. Consequently, both the photophoretic and drag forces are significantly smaller than the friction force (μN), thereby negligibly contributing to the spiral motion.

To confirm that the surrounding air is inessential to the observed spiral motion, we repeat experiments in a vacuum chamber. As expected, we observe that gold plates can also spirally move around microfibers in vacuum, as is shown in Fig. S19.

9. SURFACE TOPOGRAPHY OF GOLD PLATES

Figure S20 gives CLSM images of two golds plate before and after that they are peeled off from a glass substrate by the tapered fibers and then are dragged to new positions on the substrates. The images

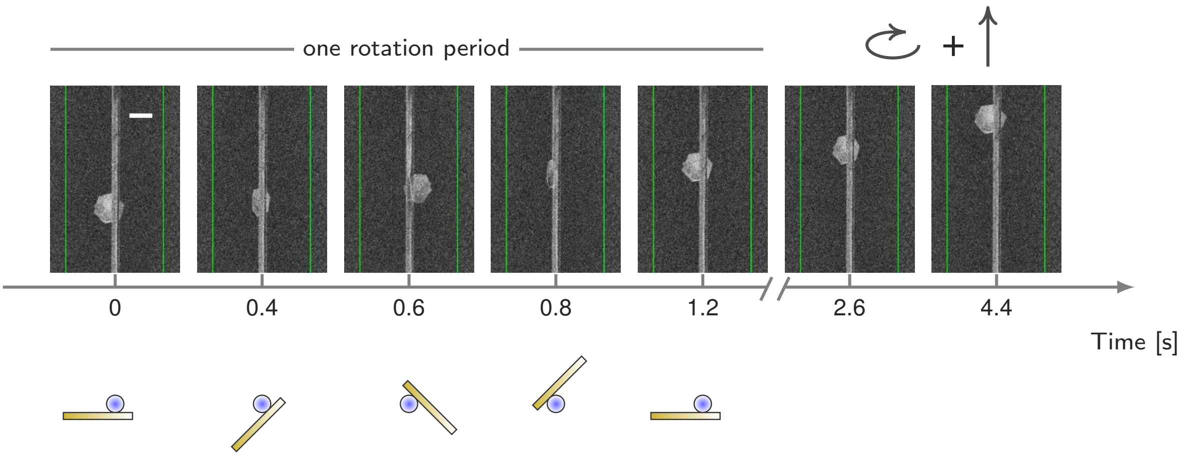

FIG. S19: **Sequential scanning electron microscopy images recording the spiral motion of a gold plate in vacuum**. Scale bar: 10 μm.

show that the plates inevitably becomesmore curved after the peeling-off process, a necessary step of transferring gold plates from glass substrates onto micro-fibers.

Figure S21 shows a confocal laser-scanning-microscopy image (CLSM) of a hexagonal gold plate on a micro-fiber, **supplementing Fig. 4F in the main text**. The image shows that the surface of the plate is apparently curved, thereby resulting in that the contact surface between the plate and micro-fiber only occupies part of the ideal one when the plate is flat.

References

[1] L. D. Landau, L. P. Pitaevskii, A. M. Kosevich, and E. M. Lifshitz, *Theory of Elasticity* (Butterworth-Heinemann, Oxford, England, UK, 2012).
[2] J. Lu, H. Yang, L. Zhou, Y. Yang, S. Luo, Q. Li, and M. Qiu, Phys. Rev. Lett. **118**, 043601 (2017).
[3] G. Batchelor, *An introduction to fluid dynamics* (Cambridge University Press, Cambridge, England, UK, 1967).

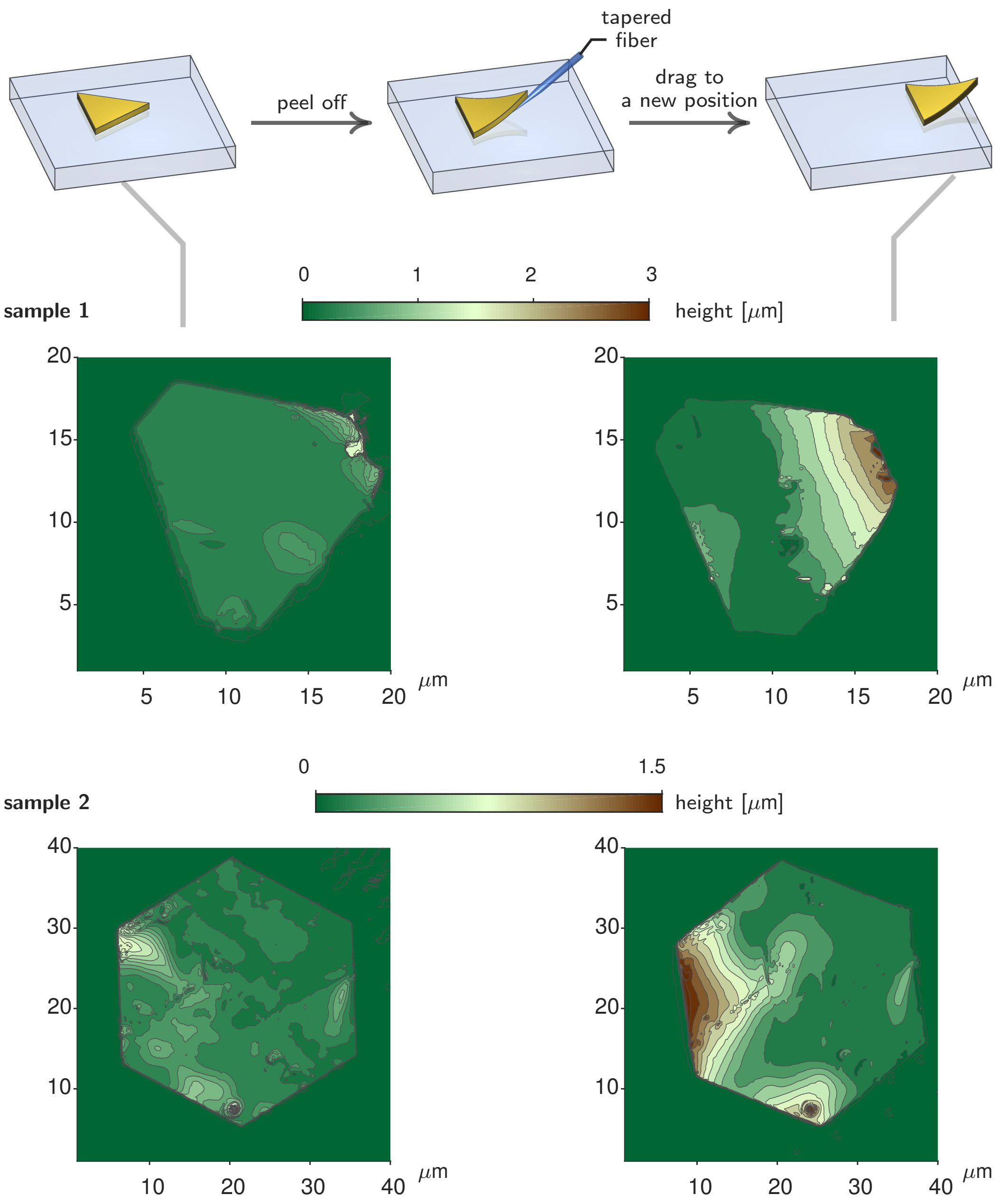

FIG. S20: **Confocal laser scanning microscopy images of two exemplified gold plates** before and after that they are peeled off from glass substrates by tapered fibers and then dragged to new positions.

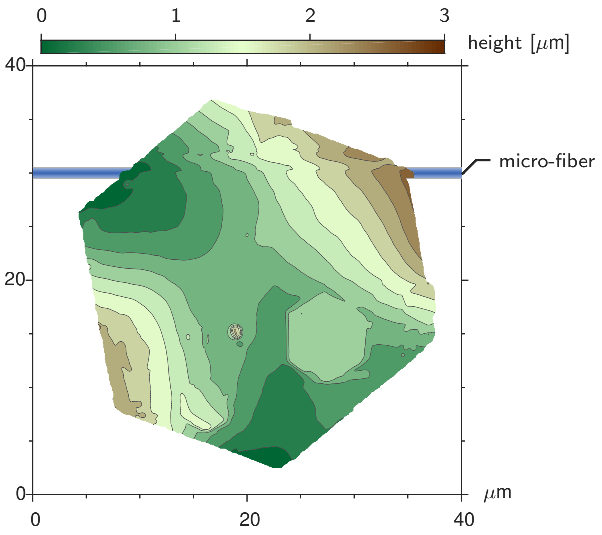

FIG. S21: **Confocal laser scanning microscopy image of a hexagonal gold plate on top of a micro-fiber**.
